# What is the role of histone H1 heterogeneity? A functional model emerges from a 50 year mystery

**DOI:** 10.3934/biophy.2015.4.724

**Published:** 2015-11-16

**Authors:** Missag Hagop Parseghian

**Affiliations:** Rubicon Biotechnology, 26212 Dimension Drive, Suite 260, Lake Forest, CA, 92630, USA;

**Keywords:** linker histones, histone H1, H1 subtypes, chromatin epigenetics

## Abstract

For the past 50 years, understanding the function of histone H1 heterogeneity has been mired in confusion and contradiction. Part of the reason for this is the lack of a working model that tries to explain the large body of data that has been collected about the H1 subtypes so far. In this review, a global model is described largely based on published data from the author and other researchers over the past 20 years. The intrinsic disorder built into H1 protein structure is discussed to help the reader understand that these histones are multi-conformational and adaptable to interactions with different targets. We discuss the role of each structural section of H1 (as we currently understand it), but we focus on the H1’s C-terminal domain and its effect on each subtype’s affinity, mobility and compaction of chromatin. We review the multiple ways these characteristics have been measured from circular dichroism to FRAP analysis, which has added to the sometimes contradictory assumptions made about each subtype. Based on a tabulation of these measurements, we then organize the H1 variants according to their ability to condense chromatin and produce nucleosome repeat lengths amenable to that compaction. This subtype variation generates a continuum of different chromatin states allowing for fine regulatory control and some overlap in the event one or two subtypes are lost to mutation. We also review the myriad of disparate observations made about each subtype, both somatic and germline specific ones, that lend support to the proposed model. Finally, to demonstrate its adaptability as new data further refines our understanding of H1 subtypes, we show how the model can be applied to experimental observations of telomeric heterochromatin in aging cells.

## 1. Introduction

May I be so bold as to suggest that neither Kinkade nor Cole would have imagined the lack of clarity that would still exist for histone H1 heterogeneity 50 years after publication of their fractionation method for H1 subtypes extracted from calf thymus [1]. The year 2016 marks the 50^th^ anniversary since Kinkade and Cole’s publication and in those 50 years the purpose of H1 heterogeneity has continued to confound researchers as they are confronted by a series of contradictory observations from the laboratories of their colleagues. It is only in the recent past a consensus has developed that H1 subtypes have functional roles, albeit some may have redundant roles, and what was once referred to as “microheterogeneity” to describe the limited number of subtypes is not merely a manifestation of genetic drift and inconsequential to the overall function of H1 [2–7]. In this review, I wish to highlight key papers, most published in the past two decades, that lend significant support to a global model of H1 subtype functionality first proposed in 2001 [2]. While a large body of important work on the H1 subtypes found in somatic cells (H1.1 through H1.5) was conducted in the latter half of the 20^th^ Century, a rather comprehensive review of that research has been published elsewhere and the reader is encouraged to refer to it for an undestanding of the groundbreaking work that has led to more recent discoveries [2].

H1 histones are one of five histone families, four of which (H2A, H2B, H3 and H4) are referred to as “core” histones as an acknowledgement of their role in forming a nucleosome core particle, an octameric structure constructed with two histones from each family that is wrapped by 146 base pairs (bp) of DNA in nearly two left handed turns [8]. Members of the H1 histone family are differentiated from the other four families by their interaction with the outside of the nucleosome, where they are situated at the site of DNA entry and exit from the core particle (known as the “dyad”) and bind a further 19–20 base pairs of DNA linking one nucleosome to the next; hence, H1 histones are often also referred to as “linker” histones. Electron cryomicroscopy studies of chromatin isolated from chicken erythrocytes indicate H1s hold linker DNA segments entering and exiting the nucleosome in apposition to each other for 3–5 nm at a point starting 8 nm away from the dyad axis [9]. This gives H1-containing chromatin the appearance of a stem-like structure interconnecting one nucleosome to the next. More importantly, this stem motif allows for the addition or removal of H1 without perturbation of the topology necessary to wind the DNA around the core histones and, therefore, rapid changes in chromatin compaction can occur without disruption of DNA packaging [9]. Such an elegant model is in harmony with chromatin conformational changes that are observed as extended nucleosomal arrays are partially folded into a “zig-zag” structure, and then into a fully condensed 30 nm fiber, with increasing salt concentrations [10,11,12]. What is lacking in this description is any mention of the H1 subtypes and their unique interactions with the nucleosome model described. That book is still being written.

When comparing the state of our knowledge about H1 subtype function to the core histone subtypes, one is immediately struck by the advancements being made to elucidate the role of core histone variants in chromatin dynamics. The dichotomy can be partially explained by the existence of a global working model for the core histones known as the “histone code” [13]. The histone code theory posits that post-translational modifications of histones, including H1s, epigenetically encode information on nucleosomes that recruit other proteins to help modify chromatin structure. Researchers today can compare their laboratory data to this global model of histone functionality allowing them to understand how their results complement or contradict a broader picture of chromatin dynamics. In this way, the histone code model is itself a dynamic theory that is modified as new information is uncovered. A similar global model that links H1 subtype distribution to each subtype’s functionality may prove useful for linker histone researchers [2,14,15]. I hope the reader comes away from this discussion in agreement.

## 2. The Challenges of Linker Histone Research

It is important to mention the reasons why the mystery of H1 subtype functionality has endured so long. First, isolation of the individual subtypes from tissue samples was a challenging endeavor due to most subtypes possessing a conserved central globular domain structure and lysine-rich primary sequences. These challenges were not overcome until the advent of gene isolation techniques and subsequent genome sequencing allowed for rapid identification of H1 family members [16,17,18]. Consider the fact that in the 1960s and 1970s we knew there was heterogeneity because of partial chromatographic resolution of some subtypes [19,20], including a highly variant avian subtype, H5 [21], and its mammalian homolog, H1° [22]. However, it was not until 1979 that electrophoretic studies by Seyedin and Kistler clearly identified five conserved somatic subtypes (H1a through H1e) and a testis specific variant named H1t [23,24,25]. The list of subtypes found in mammalian cells continued to grow with the discovery of another somatic subtype, H1x in 1996 [26], an oocyte-specific subtype, H1oo in 2001 [27], a sperm-specific subtype, HILS1 in 2003 [28], and a spermatid-specific subtype H1T2 in 2005 [29]; bringing the current total to 11 H1 subtypes in mammals discovered over the course of 40 years.

Second, several research groups assigned their own nomenclature to the same proteins based on their different methods of isolation and analysis resulting in no less than 12 different nomenclatures for the somatic subtypes alone by the end of the 20^th^ Century. This led to confusion among researchers as they tried to correlate results across laboratories. Attempts at harmonization began with the proposal of a single nomenclature in 1994 [30] with researchers eventually settling on the nomenclature developed by the Doenecke lab [18]. Table 1 correlates the three most relevant nomenclatures used in the papers discussed here in order to help readers who want to consult the references further.

Third, the apparent simplicity of H1 protein structure, replete with basic amino acid residues that bind the negatively-charged phosphate backbone of DNA, disguised the pleiotropic nature of these proteins, leaving researchers with the initial impression that these histones were general repressors of gene transcription [31,32] and DNA replication [33] by inhibiting access of transcription factors [34] and replication machinery [35] to their respective binding sites through H1-mediated chromatin condensation. While this is true for some subtypes, we now know members of the H1 protein family have roles not only in stabilizing chromatin structure [36], but also in specific gene regulation, cell signalling and immunology (for a comprehensive review of histone proteins in cell signalling and immunology see [37]). Therefore, it would not be surprising for a multi-functional protein family to have some members evolve redundant roles to counter the effects of mutations on the long term survival of the organism. Early work with H1 subtype knockouts having uncovered this built-in redundancy suggested H1 heterogeneity had no functionality [38,39,40], however, upon further analysis, researchers began to glimpse important roles once they knocked out certain combinations of subtypes and saw interesting phenotypes that could not be alleviated by the redundancy [5,6]. Only now are we beginning to understand how these low molecular weight (~20–22 kD) protein structures cause their pleiotropic effects.

## 3. H1 Structure: A Conserved Globular Domain Shows More Functionality than Once Thought

**Figure 1.**
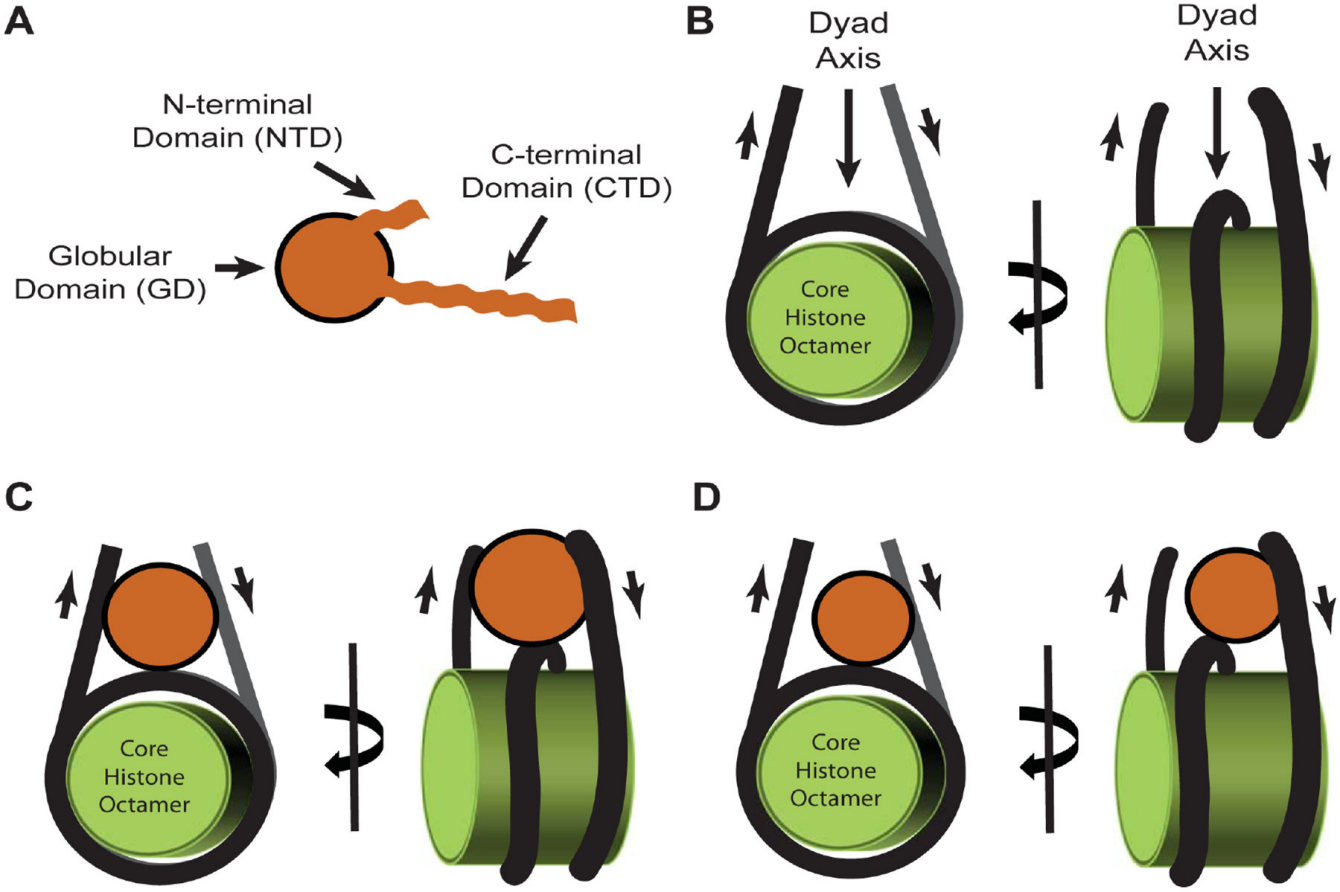
Symmetric Versus Asymmetric Models of Linker Histone Binding. (A) A stylized image representing the tripartite structure of histone H1, including the winged helix globular domain and the intrinsically disordered N- and C-terminal tails. (B) The nucleosome consists of the core histone octamer constructed from pairs of H2A, H2B, H3 and H4 histones and wrapped by 1.75 left handed turns of DNA. The dyad axis is the halfway point for the 146 bp of DNA that wrap around the octamer. (C) In a symmetric model of H1 binding, the globular domain interacts with DNA at the center of the dyad and with linker DNA entering and exiting the nucleosome. (D) In an asymmetric model, the globular domain is positioned away from the center of the dyad and interacts with only one linker DNA.

H1 histones have a tripartite division consisting of a structured central globular domain (~80 residues) and two apparently random coil tails at the amino- and carboxy-termini [41,42]. For this review, we will use a stylized image to represent the tripartite structure of the molecule (Figure 1A). The globular domain (GD) structure is well conserved between species and across subtypes as evidenced by sequence comparisons (e.g. [43]) and structural studies using X-ray crystallography (avian H5 [44]) and nuclear magnetic resonance (avian H1 [45]). However, conserved does not mean identical and only the subtypes H1.1, H1.2, H1.3, H1.4 and H1.5 have nearly identical globular domain sequences. The remaining subtypes have differences in residues at key sites when comparing their primary sequences to the canonical GD of H1.1–H1.5 [43].

The globular domain consists of three α-helices and three β-strands, one strand located between helices I and II and the others located carboxy-terminal to helix III. These last two β-strands run anti-parallel to each other and interact with the first strand to form a β-sheet in the middle of the globular domain surrounded by the three helices. In recent publications, researchers have backed away from calling the link between helices I and II a β-strand [46,47]. It is designated as segment L1, while the region linking helices II and III is referred to as L2. A β-loop located between the last two β-strands had the appearance of a wing to those individuals elucidating the structure, hence their description of the globular domain as a “winged helix” fold [44]. This wing is now designated as L3 [46].

Based on *in vitro* studies using staphylococcal nuclease digestion of the DNA surrounding assembled nucleosomes, the GD was once considered solely responsible for the positioning of H1 proteins at the dyad axis of the core particle where it alone, without the need of its tails, can complete wrapping of the 1.75 turns of DNA to a full 2 turns [41] (Figures 1B, C). These studies were refined down to a single base pair resolution of H1 interaction with nucleosomal DNA using hydroxyl radical footprinting 30 years after the original work; the new research team verifying *in vitro* that the GD interacts with 10 base pairs of the DNA minor groove at the nucleosome’s dyad axis and with one helical turn of both the linker DNA entering and exiting the nucleosome [48]. The latter studies confirmed earlier computer modelling of the GD interaction with the core particle [49] and conform with electron cryomicroscopy studies describing a stem motif for linker DNA between nucleosomes [9]. However, the story did not end there given contradictory observations by others that left open the question whether H1 binds the nucleotides in the center of the dyad, as well as the linker DNA entering and exiting the nucleosome, in a symmetric manner (Figure 1C). Some suggested the binding occurs in an asymmetric manner, with the GD positioned away from the center of the dyad and interaction with only one linker DNA [50–52] (Figure 1D). It now turns out both models are correct and the positioning of the globular domain symmetrically or asymetrically is subtype dependant.

Comparing the nucleosome binding affinity of wildtype H1° and a series of point mutants, one team identified two distinct sites on the globular domain: Site 1, containing helix III, interacts with the DNA major groove near the nucleosome dyad and Site 2, consisting of residues from more flexible domains, interacts with one strand of linker DNA [52]. This asymmetric positioning of the GD within the nucleosome dyad is independently corroborated by nuclear magnetic resonance (NMR) studies using *Drosophila* H1 [46] and cryogenic electron microscopy studies using human H1.4 [53]. However, the exact location of Sites 1 and 2 on the GD itself differs for H1° [52] compared to H1.2 [54] and H1.4 [53] due to differences in primary sequence at key residues. One would expect that H1.5 would have a similar asymmetric interaction with the dyad given its nearly identical globular domain sequence compared to H1.2 and H1.4, and yet, hydroxyl radical footprinting places the H1.5 GD precisely at the center of the dyad, binding the DNA minor groove rather than the major one, and interacting with ~ 20 base pairs of linker DNA both entering and exiting the nucleosome [48]. This symmetric interaction with the dyad may be similar to avian H5, although sequence differences would suggest the exact residues on the surface of it and the H1.5 globular domains that are interacting with the dyad and linker DNAs will be different. In the case of H5, the symmetric interaction with the nucleosome results in the GD being positioned such that helix II, not helix III, is binding the dyad [47]. Helix III binds one linker DNA and the L1 segment binds the other, with the H5 GD bringing the two linker DNAs closer to the dyad by ~ 10 Angstrom. How can the GD show such variation in its placement at the nucleosome dyad axis? The answer appears to lay with the random coil tails flanking both sides of the globular domain.

## 4. H1 Structure: A Deceptively Simple Structure of Random Coils

The amino- and carboxy- terminal domains (NTD and CTD, respectively) are deceptively simple in sequence, largely consisting of alanines, prolines and lysines throughout the 40–50 amino acids making up the NTD and the ~ 100 residues of the CTD. Both tails were considered random coils with no structural signficance until work in the Suau lab uncovered inducible structural elements. The NTD is itself a bipartite structure with hydrophobic residues in the amino-terminal half and highly basic residues in the carboxy-terminal half [55]. Once placed into a secondary structure inducing stabilizer, like 90% trifluoroethanol (TFE), the NTD of subtype H1° formed a non-amphipathic α-helix [55] and the NTD of H1.4 an amphipathic one [56] as confirmed by circular dichroism (CD) and nuclear magnetic resonance (NMR) analyses. Using infrared spectroscopy (IR), the researchers also demonstrated the 6–7% helicity of the NTD in water increased to 20–30% upon binding of this H1 fragment to DNA, a helicity comparable to that seen with TFE using both IR and CD. Such observations suggest the NTD may have a structural role in stabilizing H1 binding to DNA, however, it’s interaction with chromatin structure still needs to be elucidated and its function remains largely unknown.

One promising avenue of inquiry suggests the NTD helps localize the subtypes. Using a GFP-fused deletion mutant of H1oo lacking its CTD, it has been shown that the GD and NTD is sufficient to mimic the wildtype subnuclear distribution of this H1 variant in oocytes and somatic cells [57]. When the CTD of H1° was fused to the H1oo NTD and GD, the subnuclear localization of this hybrid histone was no different than wildtype H1oo. At least in the case of the oocytic H1 subtype, the NTD, in concert with the GD, helps control the H1oo’s location. For other subtypes, it appears to play a critical role in binding affinity. Swapping the NTD of H1° for H1.2 results in a hybrid protein with an affinity for chromatin similar to H1.2 and exchanging the H1.2 NTD for H1° produces an H1°-like affinity [58]. A second promising inquiry suggests the NTD helps mediate interactions between the H1 globular domain and other proteins. Researchers covalently linked H1° to beads and incubated them with four different human cell line extracts before characterizing the coprecipitated proteins using mass spectrometry [59]. Of the 107 binding proteins identified, 75% were isolated using an H1° deletion mutant lacking the CTD. Many of the proteins were splicing factors or involved in ribosome function and translation and rRNA synthesis and processing. Since the NTD takes on a helical structure upon binding DNA, it would be interesting to see what proteins are coprecipitated when H1° is in this state. One might envision the possibility that asymmetric binding of the globular domain in the dyad axis accommodates a non-histone protein that is bound either to the NTD, the GD, or both.

Compared to the NTD, the CTD is now the focus of much research, most of which I will not revisit here since an excellent review of the H1 carboxy tail has already been published and is worth reading [60]. Like the NTD, the CTD has a helical structure, which was first identified in sea urchin sperm H1 fragments suspended in the secondary structure inducing stabilizer, sodium perchlorate, and analyzed by CD [61]. Researchers in the Suau lab have extended this work suspending a fragment of the mammalian H1° CTD adjacent to the globular domain (fragment “CH-1”, residues 99–121) in 90% TFE to verify the helical structure using CD and NMR [62]. They then studied the same fragment’s interaction with DNA using IR spectroscopy and noted near total replacement of the random coil with amphipathic α- and 3_10_ helical structures [63]. The amphipathic helices had all the basic residues on one side of the helix and all of the hydrophobic residues on the other and while the basic residues are expected to interact with the DNA phosphate backbone, the nature of the hydrophobic patch on the other side and its possible interaction with another protein within the nucleosome structure is unclear at this time. Further IR investigations with the entire CTD domains of H1° and H1t bound to DNA revealed both sequences became fully structured in physiological salt (140 mM) with 24% of each sequence in the form of an α-helix, 25% as β-strands and the remainder as structured turns, loops and possibly 3_10_ helices [64]. The induced secondary structure acquired by the CTD in 140 mM salt was found to be stable up to 80 °C [64]. Such a stable structure could explain why the CTD has now been recognized as the main determinant of an H1 subtype’s affinity for chromatin [65,66], as well as the H1 domain responsible for mediating the stem motif within the nucleosome, stabilizing locally folded chromatin structures and assisting in the self-association of nucleosomal arrays constructed *in vitro* [67]. The CTD also may play a role in the inhibition of DNA replication [68]. Last but not least, while the NTD affects the subnuclear distribution and binding affinity of the GD to chromatin, the CTD influences the orientation of the globular domain and helps determine which surface sites will interface with the nucleosome dyad [58]. So the variation in CTD primary sequence seen among the H1 subtypes has an effect on the different positionings at the dyad axis of the more conserved globular domains. How the CTD influences the GD is still an open question.

## 5. Intrinsic Disorder

The myriad of roles managed by the CTD with what appears to be a random set of prolines, alanines, lysines and a few serines, threonines, glycines and valines can only be partially explained through a charge neutralization mechanism in which the CTD merely sequesters the negatively charged DNA backbone, hence creating a local environment conducive to chromatin compaction. We must start thinking of the CTD and the NTD as multi-conformational structures whose form is dictated by whatever target they interact with, such that the structure of the domain may change significantly whether interacting with DNA, another histone, or with another protein entirely. This lack of a well-defined conformation under native conditions is referred to as “intrinsic disorder” [69]. The stable helical structures observed for the CTD binding to DNA [64] may be entirely different secondary structures upon CTD binding to the H1 chaperone protein NASP [70] or the chromatin-remodelling protein prothymosin α [71]. Intrinsic disorder does not contradict a charge neutralization mechanism for H1 binding to DNA, it simply suggests that charge neutralization is one mechanism among others that may be employed by the H1 proteins in their interactions with other biological structures. Intrinsic disorder allows us to understand how the CTDs of H1° and H1t both displayed similar structural motifs upon binding DNA in 140 mM NaCl despite only having 30% sequence identity [64]. The primary sequence structure between H1° and H1t is not conserved, yet the secondary structure is conserved based on similar amino acid compositions between the two CTDs resulting in the induction of identical structural motifs when both proteins interact with DNA. This could explain how somatic H1 subtypes possessing variable CTD sequences can, *in vitro*, stabilize chromatin fibers in extensively folded states and facilitate nucleosome array self-association to similar extents [72]. It could also explain how the same subtypes can each have their CTDs equally activate the apoptotic nuclease DFF40 [73]. And it could explain how all of the somatic subtypes, H1.1–H1.5, as well as H1° and H1t, can have a strong selective preference for a specific AT-rich DNA sequence (known as the “A-tract”) in scaffold-associated regions (SARs) [74].

To understand whether the entire CTD is one intrinsically disordered unit or domains exist within the carboxy terminal tail, four H1° deletion mutants beginning from the C-terminus were created, including 24, 48, 72 and 97 residue deletions, the last one eliminating the CTD completely and leaving the H1° NTD and GD [67]. Using an *in vitro* model of reconstituted nucleosomal arrays, researchers were able to determine that a 24 amino acid subdomain immediately C-terminal to the GD (residues 97–121) was sufficient to mediate a stem motif with the linker DNA, stabilize locally folded chromatin and oligomerize the nucleosomal arrays. A second discontinguous subdomain (residues 145–169) was also capable of stabilizing folded chromatin. Deletion of either subdomain resulted in loss of the identified functions, whereas deletion of the other two subdomains had no effect on H1° functionality in this assay [67]. In a follow-up study, the subdomain closest to the GD (residues 97–121) had its amino acid sequence randomly scrambled while still maintaining the same composition of residues. The CTD still bound to linker DNA and stabilized the condensed chromatin fibers in the reconstituted nucleosomal arrays, lending further support to the intrinsic disorder model [72]. Note that this is the same sequence referred to by Suau’s group as the CH-1 fragment, which they showed through IR spectroscopy takes on a helical structure upon binding DNA [63]. Finally, before one gets the idea that the CTD and its subdomains are autonomous from any spatial position effects, the most surprising observation in this follow up study was the shuffling of the subdomains and the creation of H1° mutants with the other subdomains replacing the wildtype sequence adjacent to the GD. In each case, the new subdomains acquired all of the stabilizing characteristics of the wildtype amino acids located at residues 97–121 [72], highlighting the important role the globular domain plays as a structural reference point in the overall protein structure.

This result, along with the observation that the CTD influences placement of the GD at the dyad axis, suggests each domain contributes to an overall H1 structure whose functionality is greater than each of its parts. This synergy has been studied by Stasevich et al. [75], whose analysis of chromatin binding affinity using mutant H1° constructs either lacking the CTD and/or posessing mutations in Site 1 or Site 2 of the GD confirms a kinetic interaction model for H1 first proposed by Brown et al. [52]. In this model, the CTD first binds the linker DNA non-specifically, placing the globular domain within the dyad axis. Either Site 1 or Site 2 on the GD then binds the dyad axis symetrically or asymetrically depending on the subtype. Binding of either site increases the cooperativity of binding to the nucleosome for the second site. Once the GD is in place, Brown et al. suggest it influences the CTD to take on a structural form that causes chromatin condensation. This is the first demonstration that H1 binding may occur in stages and opens up the possibility that H1 interacts with other proteins during states of intermediate binding to the nucleosome.

## 6. Post-Translational Modifications: Further Diversifying the Functional Variation of H1 Subtypes

No discussion of structure would be complete without mention of H1 post-translational modifications, which are now receiving greater attention from the mass community [76]. The one modification that has dominated this area of research is phosphorylation given the early identification of cAMP-dependent [77] and cyclin dependent kinase (CDK) [78] sites, the latter recognizable by the presence of a S/T-P-X-K/R tetrapeptide motif. These sites are located in the NTD and CTD and vary in number and location depending upon the H1 subtype [30,79]. The serine or threonine is phosphorylated resulting in disruption of the H1 binding to the DNA [65]. These conserved sequences co-exist in the intrinsically disordered tails of H1, where they take on a distinct β-turn structure upon contact with the DNA minor groove [62,80]. Vila et al. (2000) suggest α-helix binding of the major groove and β-turn binding of the minor groove provide greater stability to the CTD upon DNA contact [62]. While electrostatic repulsion of the H1 from the DNA backbone is the likely result when all sites are phosphorylated during mitosis, Lopez et al. (2015) wanted to understand the effects of partial phosphorylation on the CTD [81], since a significant proportion of H1s are in this state during interphase and particularly during S-phase [82]. In fact, we now know that phosphorylation of the sites on an H1 molecule are not random, with serine phosphorylations occurring during interphase and threonine phosphorylations occurring during mitosis [79]. Using IR spectroscopy, they determined partial phosphorylation decreased α-helical and β-turn structures on the CTD while increasing the β-sheet conformation. Since 70% of the lysine residues in the CTD are in doublets [81], they propose the folding of the intrinsic domain into β-sheet structures results in adjoining lysines projecting in opposite directions, thus decreasing the level of charge neutralization along the DNA backbone [81]. In their model, a decrease in the net positive charge by the addition of the phosphate group to the H1 is compounded by the decrease in electrostatic potential caused by the neutralization of the adjacent lysines.

Similar analyses with other post-translational modifications of H1 are warranted in order to understand the effects of these modifications on each of the subtype structures. We know that H1 subtypes vary in their levels of phosphorylation and poly(ADP-ribosyl)ation [79,82,83,84] (see Table 1 in [2] for a summary), it is certainly not out of the realm of possibility that they do so with respect to other modifications. We also know the effects some of these modifications have on H1 in its interactions with chromatin structure. Poly(ADP-ribosyl)ation leads to a decrease in chromatin compaction surrounding H1 [85], phosphorylation reduces H1 binding to the linker DNA, yet we are still ignorant of how these and other modifications such as methylation [86,87,88], precisely affect H1 *protein structure* either alone or in combination with other modifications. Note the 24 year gap between identification of CDK phosphorylation sites and the IR studies describing H1 structural changes during phosphorylation. Case in point, with the presence of multiple CDK phosphorylation sites in the CTD, one would envision an incremental decrease in binding to the DNA as each site is modified, so it was surprising to find that phosphorylation of only threonine-152 in the CTD of subtype H1.1 was sufficient to destabilize binding of the protein to DNA *in vivo* [65]. Phosphorylation of this single site resulted in an H1.1 as ineffective in its binding to chromatin as a truncation mutant from residue 151 to the C-terminus [65]. We may now start to understand why serines are phosphorylated in interphase and threonines during mitosis, a time when some subtypes may be completely removed from the compacting chromosome. And maybe now that we know H1 proteins acquire multiple conformations, identifying those structures and seeing how they change during post-translational modifications will help us better understand suprising results such as the phosphorylation of threonine-152.

The need to study native and modified H1 structure using IR and similar methods also has important implications on the quality of research currently being conducted. If a single phosphate moiety can bring about such significant structural changes to a histone, what are the structural changes we are unaware of when we fuse fluorescent proteins [89], Dam methyltransferases [90] and FLAG tags [91] to the NTD or CTD? One research team determined that a GFP fusion to the histone H1 C-terminus influenced binding to chromatin, so all of their subsequent studies were conducted with GFP fusions to the N-terminus [89]. Most researchers often compare the binding characteristics of their fusion molecules to the wildtype version, if data exists for the wildtype; however, that may no longer be enough given the unforseen changes that can occur in a protein that is characterized by intrinsic disorder. Researchers should be expected to compare the IR profiles of their constructs to the wildtype versions while both molecules are bound to DNA in order to determine if the fusion of tags or proteins significantly changes the percentage of helices, sheets and turns that make up the dynamic structure of an H1.

## 7. Nomads of the Nucleus

Perhaps a fourth reason why our understanding of H1 functionality has lagged behind that of the core histones is the simple fact that H1 histones just don’t sit still. This mobility has consequences beyond the nucleus given the role H1s, and some core histones, play in cell signalling and immunology [37], but for this discussion we will stay within the nuclear realm. Core histones are *relatively* immobile compared to their linker histone counterparts, an observation first made in 1974 when researchers created “heterokaryons” fusing HeLa cells with inactive chick erythrocytes [92]. In a series of pulse-chase experiments, HeLa cells were radiolabelled and the migration of human proteins, prominent among them the H1 histones, were tracked as they populated the chick erythrocyte nucleus. Concurrent with this migration, the compacted chick chromatin decondensed and reactivated protein transcription, which had, until that point in time, shut down *de novo* synthesis of avian proteins. As we will see shortly, not all H1 subtypes repress transcription equally and the phenomenon witnessed in these early experiments can be explained by the replacement of the highly repressive avian histone H5 with human counterparts less able to condense chromatin as effectively. This work was the forerunner of several independent studies that have looked at the ability of oocyte specific H1s to swiftly alter global distributions of other H1 subtypes as part of the process of nuclear reprogramming when exogenous chromatin enters an egg, as in the case of sperm nuclei during fertilization or somatic nuclei during a nuclear transfer experiment [93]. Reacquisition of transcriptional and replicational competence following replacement of somatic linker histones with an oocytic counterpart (H1oo) occurs whether working with a *Xenopus* [35,94] or model [93,95] and highlights an example of an H1 subtype that binds chromatin with greater affinity than some other subtypes while simultaneously exhibiting minimal chromatin compaction. But how can one quantitate this affinity *in vivo*?

The mobility of a subtype across the nucleus is somewhat nomadic as many histone molecules are in a state of dynamic interaction with chromatin, binding a nucleosome temporarily before migrating to a new site [96]. At any given moment in time, the majority of H1 subtypes would be bound to the chromatin in a steady state [97], however, different subtypes may possess different residence times, some subtypes bouncing on and off H1 binding sites more frequently than others. In this way, one can envision subtypes with varying affinities for chromatin modulating the access of transcription and replication factors at two levels: first, at the higher-order chromatin level they stabilize compaction and second, at the level of the individual nucleosomes they can sterically block access to other chromatin binding proteins [98]. One ingeniuous method for observing this phenomenon and using it to measure chromatin binding affinity requires GFP-fused histone proteins be transfected into a cell line and stably expressed. It is incumbent upon those conducting these studies to demonstrate that histone expression is comparable to the wildtype cells in order not to cause perturbations in the chromatin structure. GFP fluorescing nuclei can then be observed microscopically and precise subnuclear regions photobleached. Within a matter of seconds or minutes, migration should repopulate the photobleached areas with the GFP-fused histones, a procedure aptly named fluorescence recovery after photobleaching (FRAP). Subtypes with a greater affinity for chromatin will have longer residence times and this translates into slower migration times across the nucleus, hence, nuclei possessing GFP-fused versions of these subtypes will take longer to recover fluorescent signal, while variants with less affinity for chromatin will take shorter amounts of time to achieve recovery. Affinity is then quantified based on the time it takes to recover the signal and is often presented by different research groups either as time to achieve 50% signal recovery (t_1/2_ or t_50_) (e.g. [97,99]), 90% signal recovery (t_90_) [99], or as the total recovery time [97] (Table 2). When plotted graphically, recovery of signal intensity often displays a biphasic curve with a rapid increase toward near full recovery followed by an asymptotic approach to 100% recovery over time. This phenomenon is explained by the fact that some histones may have a portion of their population locked into an immobile state, perhaps interacting with other chromatin binding proteins, which impedes recovery to a full 100%. Thus, t_1/2_ or t_50_ measures the affinity of the mobile fraction of proteins and total recovery helps identify the immobile fraction.

Mobility is not relegated to H1 histones, indeed, a survey of chromatin proteins with diverse functions finds that most of them have dynamic interactions with chromatin on the order of seconds [97]. Histones have a tremendous range. For instance, in a study of HeLa cells, >80% of the GFP fused versions of human core histones H3 and H4 remain bound to chromatin for several generations. Therefore, generating a FRAP analysis for either of these proteins results in a curve that plateaus far below 100% recovery, preventing the bleached area from returning to its original fluorescent intensity until several rounds of DNA replication replace the core histones with *de novo* synthesized GFP-H3 or GFP-H4. In contrast, there are three groups of H2B histones based on mobility: ~ 3% with a t_1/2_ = 6 min, 40% with a t_1/2_ = 130 min, and the rest with a t_1/2_ > 8.5 hrs [100]. H1 affinities in terms of mobility are much lower and have been determined for all but the testis and spermatid-specific subtypes; unfortunately, different researchers have not reported their findings in a uniform manner (Table 2). Just as importantly, some results are based on GFP fusions to the C-terminus, which does influence binding of H1 to chromatin [89], and others with the GFP attached to the N-terminus. Table 2 clearly illustrates this for H1°, where GFP fused to the CTD produces a hybrid molecule with much shorter t_50_ recovery times and, thus, weaker binding than H1° fused to GFP at the NTD.

The most comparable results are from the Hendzel lab (see top 3 rows in Table 2), which conducted two studies evaluating recovery times for most somatic subtypes using GFP fused to the NTD and classified the H1s into three affinity groups. In their first study, they evaluated the total recovery and determined that H1.1 and H1.2 had recovery times on the order of 1–2 min, H1° took “several minutes”, and H1.3, H1.4 and H1.5 took up to 15 min [89]. In their second study, they evaluated the t_50_ and t _90_ for each of the subtypes using 10T1/2 mouse fibroblast cells. The t_50_ provides an affinity measurement for the highly mobile variants of each subtype while the t_90_ measures the affinity of immobile or tightly bound variants in each subtype [99]. The researchers’ results were similar to their earlier study: H1.2 showed the weakest affinity for chromatin, H1.1 and H1.3 had an intermediate affinity and subtypes H1.4, H1.5 and H1° showed the highest affinity. These differences represent each subtype’s average relative affinity for chromatin and reflect their different on and off rates independent of diffusion rates given determinations that H1s diffuse through the nucleoplasm on the order of 200–400 milliseconds between binding events [97].

Affinity values can vary based on post-translational modifications to the H1. For example, the same subtypes can show differences based on phosphorylation state [101]. The FRAP method is sensitive enough to differentiate wildtype H1s from site-specific mutants. In fact, several of the affinity studies to help clarify H1 binding to the nucleosome dyad, which we have already discussed, used this method [52,54,58,75]. Affinity values can also vary given the state of the chromatin. For instance, H1 subtypes exhibit slower t_1/2_ migration times in differentiated leukemia cells than their neoplastic parental cell lines [102]. Raghuram et al. hyperacetylated the core histones in mouse fibroblast cells to study the effects on each H1 subtype’s affinity and their residence times bound to the globally decondensed chromatin [99]. While it was expected that the hyperacetylation would disrupt all of the H1 subtypes from binding the chromatin with high affinity, they actually observed differential effects that were subtype dependent, allowing them to speculate that different H1 subtypes have different requirements for binding chromatin. Case in point, hyperacetylation caused a reduction in high affinity binding of the subtypes studied with the exceptions of H1.2 and H1.5 (see Table 1 in [99]).

Looking at Table 2, there is a correlation between the length of the CTD and the affinity to chromatin as evidenced by the shorter subtypes, H1.1, H1.2 and H1x having weaker affinities compared to the longer subtypes H1.3, H1.4, H1.5 and H1oo [57,89,103]. H1° is the exception since it has the shortest CTD and yet has a strong affinity for chromatin, likely due to a higher lysine content. The CTD is the determinant of each subtype’s affinity for chromatin [65,89]. Its deletion increases H1 mobility [104]. The key driver of dissociation in somatic cells is phosphorylation at the CDK sites [101], while in oocytic cells, an entirely different mechanism exists in the form of citrullination at a single arginine residue in the NTD [105].

## 8. Measuring Affinity

Long before FRAP analysis was in vogue for measuring affinity, researchers were using *in vitro* reconstructions of chromatin to measure subtype binding affinities and aggregation capabilities to DNA and chromatin. Rather than give lengthy discussions about the reported results of each study, I have summarized them in Table 3. The reader will note that I have placed the subtypes out of numerical order in both Tables 2 and 3 with H1.5 in between H1.2 and H1.3. This reflects my contention that H1.5 is affiliated more with openly transcribed chromatin or regions poised for transcription (see below). Also, note that these studies from independent labs can be placed in one of two categories regarding the nature of the starting material: some studies use nucleosomes lacking or depleted of H1 and observe binding characteristics as a single subtype is added to the system (Table 3, highlighted in tan); other studies already have wildtype H1 present on the nucleosomes and observe the effects of adding a single H1 subtype to the system (Table 3, highlighted in green). These studies have taken on greater sophistication over time. Early reconstruction studies looked at aggregation of dinucleosomes using circular dichroism, however, they occurred at 80 mM NaCl, well below physiological salt concentrations [106]. And while some later studies have looked at differences in binding to DNA and mononucleosomes [107,108], they have been part of larger studies looking at aggregation of oligonucleosomes, a more relevant reconstruction of the nuclear environment than naked DNA or mono- and di-nucleosomes. In the case of Talasz et al. (1998), the ability of reconstituted subtypes to aggregate a well-defined MMTV LTR polynucleosome structure (1.3 kb, 6 nucleosomes) in 50 mM NaCl revealed H1.5 and H1t are much weaker and require greater amounts of protein to cause aggregation, than H1.3, H1.4, H1a and H1°, while H1.2 showed an intermediate aggregation ability between the two other groups [108]. An independent study confirmed the weak aggregation of chromatin by H1t [107]. The ability of H1a to strongly aggregate oligonucleosomes in this system was surprising given the observation by others that H1a, H1.2 and H1t are affiliated with more open chromatin conformations, as is the case in pachytene spermatocytes where all three subtypes predominate in a chromatin environment that must remain open in order to allow for meiotic recombination [12].

Perhaps an under-appreciated study is the work of Hannon et al. (1984), who used H1 depleted chromatin (20–100 nucleosomes per chain) in an 80 mM NaCl buffer to not only reconstitute oligonucleosomes with each H1 subtype available at the time, but also test their transcriptional inhibition of an *E. coli* RNA polymerase [109]. H1.2 reconstituted chromatin was so weak in its inhibition of transcriptional initiation that it took the addition of two H1.2 per nucleosome into the system to bring about transcriptional inhibition comparable to H1.5. Meanwhile, H1.5 proved to be less inhibitory than H1.4 and H1° [109]. In a similar manner, Clausell et al. (2009) tested the ability of subtypes to affect the nucleosome repeat length (NRL) and inhibit chromatin modeling [66]. They assembled minichromosomes on a 5.12 kb supercoiled plasmid containing the MMTV promoter using preblastodermic Drosophila embryo extract, which naturally lacks H1, and then added pure subtypes to study the effects. They looked at chromatin compaction in a 20 mM KCl buffer and found signficant differences, yet this variation in compaction had no effect inhibiting ATP-dependent chromatin remodeling with either SWI/SNF or NURF in a 60 mM KCl buffer. Investigating at a more physiological salt concentration may clarify the discrepancies. They also chose to define subtype affinity by determining the amount of each variant needed to generate a specific NRL on their defined chromatin fragments (see graph 1B in [66]), finding H1.5 to be the most efficient NRL generator, and therefore, the subtype with the greatest affinity. Their categorization of each subtype is similar to the cell-based observations of Th’ng et al. [89] and Raghuram et al. [99].

Several researchers have chosen not to deal with artificially reconstituted chromatin, instead choosing to change the overall proportion of one subtype over others and studying the effects on native chromatin structure. One clever approach modified the concept of FRAP into an *in vitro* assay, utilizing H1’s transient binding to chromatin to characterize each subtype’s affinity at near physiological salt concentrations. Orrego et al. (2007) chose to mix histone subtypes into a 140 mM NaCl buffer containing chromatin fragments (30–35 nucleosomes long from rat liver). Within 30 minutes, the added subtypes achieved equilibrium binding to the chromatin, the oligonucleosomes were pelleted and the amount of exogenously added subtype displacing the existing wildype H1s was determined [110]. The authors chose to compare these affinities to a SAR DNA sequence (657 bp from *Drosophila*) known to bind H1 subtypes avidly [74] and what they found is instructive: although most of the subtypes had the same relative affinities for binding chromatin or the SAR sequence (Table 3), H1.5 has a shifiting affinity which is stronger for the naked SAR DNA, but weaker for chromatin in general when compared to subtype H1.2, whose binding affinity for DNA or chromatin is low to begin with [110]. Given what we are now learning about the intrinsic disorder built into H1 structure, it is entirely possible that a subtype such as H1.5 will interact differently with a stretch of DNA depending on how it is packaged or not within the nucleus. A similar situation may be at work for HILS1, the spermatid-specific H1, which binds DNA far more avidly than it binds a nucleosomal structure when compared to the affinity of H1.1 (Table 3). As we will see ahead, HILS1 binds chromatin in a spermatid nucleus that is lacking core histones, so its differential interaction with a stretch of DNA, depending on how that DNA is being packaged, may have real *in vivo* consequences.

While reconstructing chromatin *in vitro* has provided useful information, others have chosen to change the overall proportion of one subtype over others *in vivo* and their observations have been largely supportive of the *in vitro* results tallied in Table 3. Mouse 3T3 fibroblasts transfected with expression vectors for H1.2 and H1° had very different outcomes on chromatin structure and overall gene expression, with early studies finding overexpression of H1.2 actually causing a dramatic increase in transcription levels [111] and subsequent microarray and real-time PCR (RT-PCR) studies finding upregulation of specific genes in the same modified cells [112]. The authors suspect H1.2 creates a more open chromatin structure within the nucleus and at specific gene locations, hence, the increased transcription. Overexpression of H1° caused a general repression of transcription and inhibition of G1 and S phase progression [111], but subsequent microarray and RT-PCR analyses found specific genes that were upregulated as well due to the increased presence of H1° [112]. Unlike H1.2, they do not propose an explanation for these seemingly contradictory effects. A separate research group inadvertantly conducted a similar study with H1.3 overexpression in human WI-38 fibroblasts [113]. While their original research goals were different, they end up showing overexpression of a GFP-H1.3 fusion molecule leads to severe growth inhibition and expression of senescence morphologies [113]. Finally, a more recent study tagged H1 subtypes with Dam methyltransferases and expressed them in the human IMR90 fibroblast line, finding the subtypes H1.2 through H1.5 largely having a similar distribution in the genome on exons, introns and intergenic regions but depleted in the promoter regions and regulatory elements of actively transcribed genes [90]. However, the authors also show differential distribution of subtypes when comparing entire chromosomes and reiterate that the minor differences seen for each subtype throughout the genome are expected to be biologically relevant.

## 9. The Origin and Diversification of Subtypes

**Figure 2.**
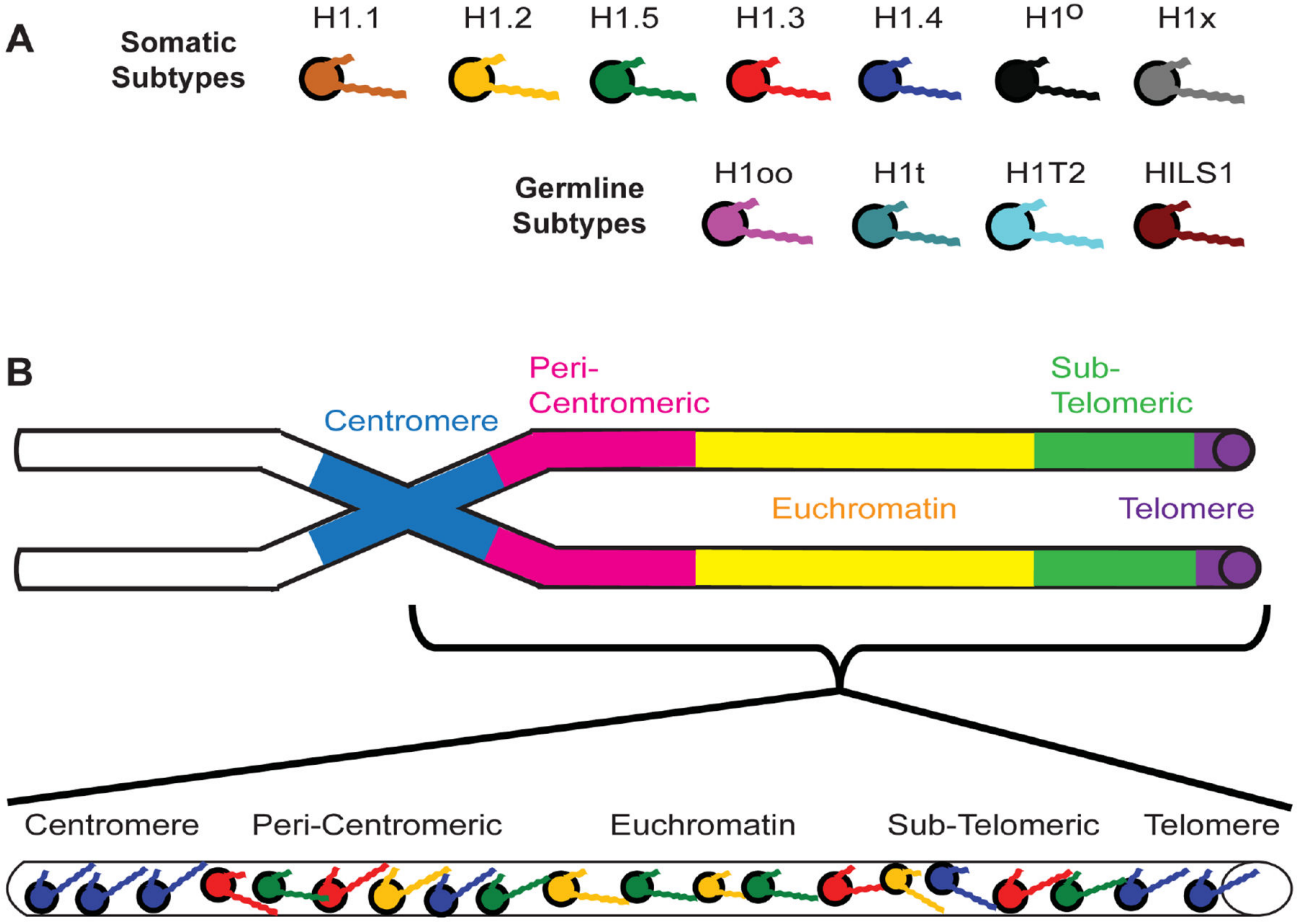
Modelling H1 Subtype Diversity. (A) The H1 subtypes discussed in the review and illustrated in the figures are each represented by a different color coded symbol. (B) A chromosome and its complex oligonucleosomal fibers are represented as a simple cylinder spanning from the centromere to the telomere.

As suggested at the beginning of this review, a working model that tries to address the global distribution of H1 subtypes would help clarify some of the disparate observations reported by researchers. A model I first proposed in 2001 [2,14,15] to help explain how chromatin immunoprecipitation (ChIP) results corroborate the work of other researchers has held up well and has been advanced by others using different analytical techniques. Perhaps the best way to summarize the model is to think in these terms: all H1s condense DNA and chromatin, some H1s condense more than others. For simplicity of illustration, I will represent each of the H1 subtypes with a different color (Figure 2A). The imagery is further simplified by representing a chromosome and its complex oligonucleosomal fibers as a simple cylinder spanning from the centromere to the telomere (Figure 2B).

The primordial cell had no need for H1 as evidenced by the lack of linker histone genes in any of the archaebacteria investigated to date [114]. While core histones originated in archaea, linker histones originated in eubacteria, with bacteria and some protists possessing linker histones essentially made of sequences similar to the CTD in mammalian H1 [114]. A prime example of this is the protist *Tetrahymena thermophila*, which possesses a single H1 in its macronucleus that is lacking the globular domain commonly found in eukaryotes [115]. At some point prior to the radiation of multicellular organisms, H1 was fused to a globular domain and an NTD, and then the subtypes H1° and H1oo diverged from the ancestral H1 gene [116]. Based on molecular evolutionary models, the divergence of mammalian somatic subtypes, along with H1t, is believed to have occurred about 406 million years ago, coinciding with the divergence of jawed vertebrates [117].

**Figure 3.**
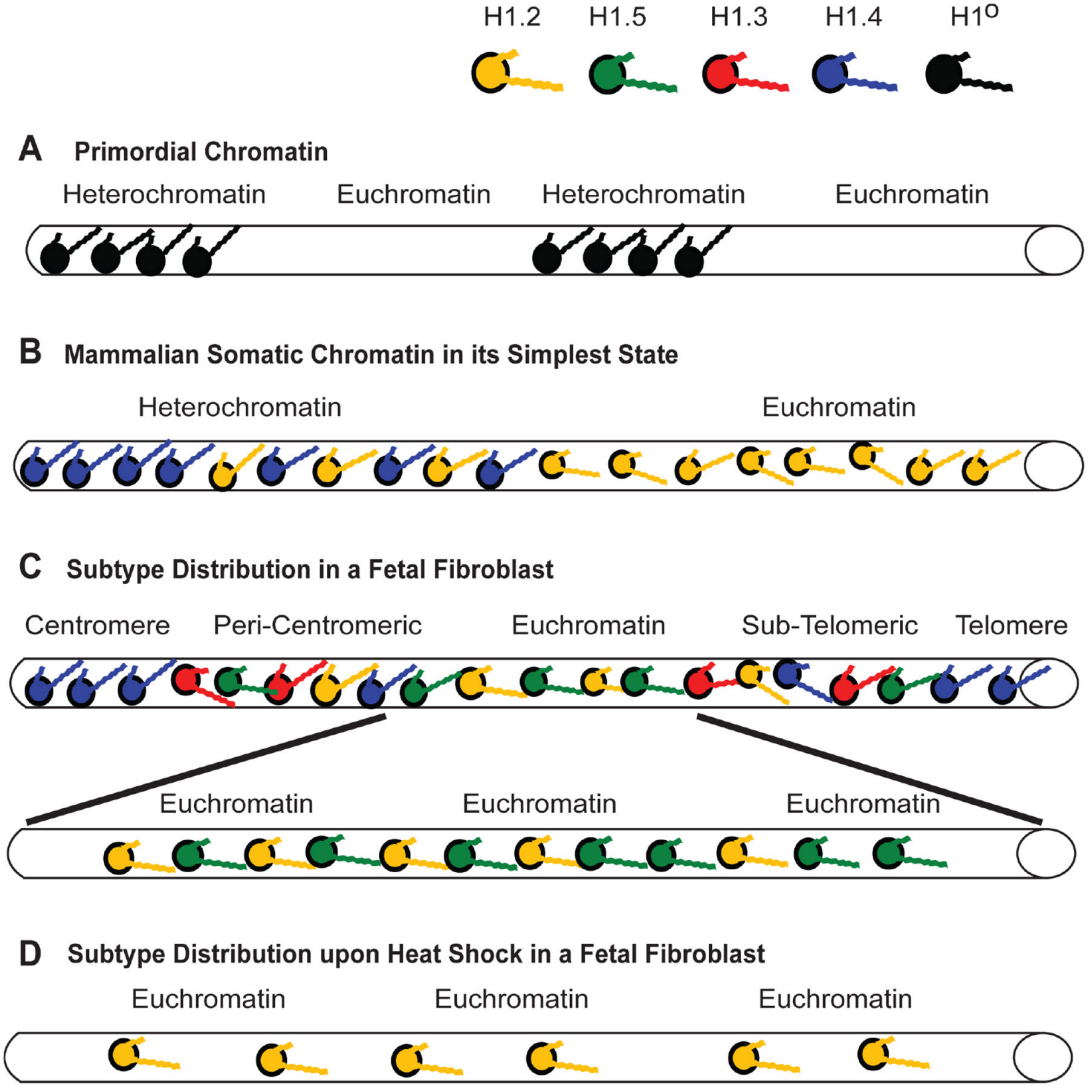
(A) Primordial chromatin. Single celled organisms have one H1 isoform, which is sufficient to generate differential chromatin compaction during development. (B) Mammalian somatic chromatin in its simplest state. Two subtypes are all that are necessary to establish broad areas of euchromatin (H1.2) and heterochromatin (H1.4) in mammalian cell culture. (C) Subtype Distribution in a Fetal Fibroblast Cell Line (GM02291). Heterochromatin and inactive chromatin have a diverse set of H1 subtypes present in human diploid fibroblast cells. Actively transcribed euchromatin is depleted of two subtypes, H1.3 and H1.4. (D) Heat Shock of GM02291 Cells. Some actively transcribed genes upregulated during heat shock also lose their association with H1.5 leaving only H1.2 bound to the DNA. In essence, this is a throw back to the somatic chromatin in its simplest state.

Based on these observations, it is easy to imagine that unicellular life forms having smaller genomes and simpler chromatin states had no need for multiple subtypes with different binding characteristics. A single primordial H1-like subtype could stabilize chromatin compaction and possibly inactivate regions of gene expression that required inhibition during the cell’s life cycle (Figure 3A). When the single H1 gene in the yeast *Saccharomyces cerevisiae* was knocked out, researchers found it detrimentally affected the cell’s life span by not suppressing excess homologous recombination. They surmised that a conserved role for linker histones is the maintenance of genomic stability through the inhibition of excess recombination [118]. Also, knocking out the single H1 genes in *Saccharomyces* and *Tetrahymena* resulted in chromatin decondensation and the down-regulation of some genes [119,120]. Whether knocking out these H1s unleashed expression of other proteins that inhibit transcription of the genes being studied is an open question. What is important to note is the multiple roles a single H1 subtype appears to take on even in single-cell organisms. The intrinsically disordered nature of the H1 CTD tail may have provided the mechanism, early in evolution, for these histones to act as pleiotropic agents.

It is not my intention to review all of the H1 knockout data published in the past 20 years. For an excellent summary of results, see Table 2 in Izzo et al. (2008) [43]. Suffice it to say, many early H1 knockout studies looked at single-cell organisms or tissue culture cells and drew conclusions that may not be relevant to the multicell complexity of vertebrates. It is important to reiterate that increasing cellular diversity in an organism born from a single genome requires greater variation in chromatin regulation, stability and compaction during development. While the number of core histone variants are generally consistent across the kingdoms of life, there is a trend toward larger multicellular organisms with diverse cell types having a greater number of linker histone subtypes (Table 4). It is also important to note that the number of subtypes and the amino acid sequences are conserved across mammalian species with man and mouse both having 11 variants after millions of years of evolution [117]. It is not my intent to ascribe the diversity of cell types solely to linker histones given the critical role played by an array of transcription factors, however, the conserved diversity of H1 subtypes, compounded by their differential levels of post-translational modifications, suggests we are only beginning to appreciate the fine adjustments to chromatin regulation that this family of proteins are capable of and their effects on long term development.

## 10. A Working Model Emerges from a Diverse Set of Chromatin Condensers

It must be said that there are multicellular organisms, presumably with less cell diversity than vertebrates, which happen to have only one subtype, case in point *Drosophila melanogaster*. In a recent study using ChIP-seq analysis, researchers determined *Drosophila* H1 is primarily associated with heterochromatic regions and gene repression [121]. So, even in some lower animals, modulation of chromatin states to only a few levels of compaction with post-translational modification of a single H1 variant is sufficient to help generate multiple cell types.

### 10.1. H1.2 and H1.4 (Parseghian’s H1^s^-1 and H1^s^-4; Seyedin & Kistler’s H1c and H1e)

The primordial state for mammalian somatic cells may have started with two major subtypes, H1.2 and H1.4, organizing the nucleus into euchromatic and heterochromatic regions, respectively (Figure 3B). Evidence of their importance comes from tissue culture experiments. In a survey of commonly used human cell lines, researchers could not find one that lacked expression of these subtypes despite identifying many cell lines with other H1s missing [122]. In studies with a human breast cancer line, knockdown of H1.4 expression with short hairpin RNA (shRNA) led to cell death, suggesting the critical role of this subtype in chromatin organization [7]. Knockdown of H1.2 led to *down*-regulation of cell cycle genes and G1-phase arrest [7].

The affinity studies summarized in Tables 2 and 3 suggest H1.4 strongly binds and compacts chromatin, making it a logical choice for promoting heterochromatinization. The affinity studies correlate well with the observation in ChIP experiments that the subtype is associated with inactive chromatin, including centromeres, in fetal [14] and adult [15] fibroblasts. Most importantly, during heterochromatin formation, SirT1 specifically recruits H1.4 and deacetylates its lysine 26 before incorporating it into the compacted chromatin structure [86]. H1.4 has a relatively slow turnover rate in neurons, where a decline in expression of other subtypes leads to its eventual predominance (~70%) [123], possibly explaining the lack of plasticity in these cells. Its interaction within regions of heterochromatin may require greater levels of phosphorylation to dislodge it from its binding site, hence, H1.4 is the only subtype known to undergo a hormone-regulated cAMP dependent phosphorylation as well as cyclin-dependent phosphorylation [77]. It also undergoes acetylation at lysine-34 to similarly promote an open chromatin conformation [124]. The critical nature of this subtype is perhaps reflected in its relatively low rate of non-synonymous amino acid mutations over millions of years, in fact it is the most conserved of the somatic subtypes [117]. The same affinity studies summarized in Tables 2 and 3 suggest H1.2 does not bind or compact chromatin to comparable levels as its somatic counterparts, H1.3, H1.4 and H1.5. Its weak binding may explain why it is phosphorylated at lower levels than other subtypes at every stage of the cell cycle [82]. As stated earlier, its overexpression *in vivo* causes an increase in transcription levels suggesting H1.2 creates a more open chromatin conformation than its counterparts [111], indeed it is enriched in soluble chromatin fractions [125,126]. The reader will note that we are still suggesting H1.2 does compact chromatin, just not at the level of other subtypes. In ChIP studies it was distributed both in active and inactive chromatin and remained associated with genes exhibiting increased transcription upon heat shock induction [14,15].

To make sense of all these observations, one must start thinking of H1.2 as a *ground state* subtype, establishing a basal level of condensed chromatin throughout the nucleus upon which other subtypes may bind to create greater levels of compaction in a cooperative manner. Those regions possessing only H1.2 have a generally open chromatin conformation that does not inhibit RNA polymerase initiation (Table 3) [109] or PARP-1 binding [127] at active promoters. In the case of PARP-1, it is important to note that H1.2 is a major target for poly(ADP-ribosyl)ation [83,128,29] despite the presence of PARP sites on all of the somatic H1 subtypes [130]. It suggests the accessibility of H1.2 to poly-(ADP-ribosyl)ation is greater than the other H1s.

With the predominance of H1.4 in heterochromatic regions, any presence of H1.2 would not disrupt the stronger compaction of nucleosomes. The deposition of H1.4 is controlled by SirT1 and by the replication dependent synthesis of H1.4, which starts in mid-S phase [131] at a time when heterochromatic DNA is being replicated. Barring any other subtypes that can replace H1.4’s functionality, its deletion from a cell would result in a general loss of heterochromatinization and disruption of gene inactivation at critical stages in the cell’s life cycle, possibly leading to cell death [7]. Conversely, H1.2 transcribes two forms of RNA, one replication dependent and the other independent [132], allowing its deposition throughout the nucleus during the entire cell cycle. Its knockdown actually inhibits expression of certain cell cycle (CDC2, CDC6,CDC23), heat shock (e.g. Hsp90α and Hsp27, genes HSP90AA1 and HSPB2, respectively) and other proteins resulting in G1-phase arrest, presumably because a more compact chromatin structure inhibits transcription of these genes to a level below what is necessary for normal cell functioning. Strikingly, H1.2 depletion with shRNA also caused a reduction in the global NRL from 184.7 to 173.5 bp [7]. As has been recently suggested, shorter NRLs not only effect compaction, but may actually disrupt the formation of a 30 nm fiber [133]. Both G1 phase arrest and the NRL were restored only when cells were transfected with an shRNA resistant H1.2, but not with any other H1 subtypes [7] suggesting the unique functional role of this subtype in maintaining global stability. Such a global decrease in the NRL from H1.2, one of the smaller H1 subtypes, lends support to a thermodynamic model proposed by Beshnova et al. (2014) in which smaller histones have a greater effect increasing the NRL due to a larger configurational entropy caused by the rearrangement of bound proteins on the chromatin [134]. However, by the authors’ own admission, this effect could only occur if the H1-DNA binding affinity did not depend on H1 size. While there is some correlation between size and affinity [89], other factors such as phosphorylation significantly affect the affinity of any subtype regardless of its size.

### 10.2. H1.5 (Parseghian’s H1^s^-3; Seyedin & Kistler’s H1b)

What of the other subtypes? ChIP experiments reveal both H1.3 and H1.5 present on inactive chromatin along with H1.2 and H1.4 [7,14,15], however, active chromatin has a significant depletion of two subtypes, H1.3 and H1.4, (Figure 3C) [14]. Once again, the affinity studies in Tables 2 and 3, particularly the work of Orrego et al. (2007) and Th’ng et al. (2005), lend support to the distribution of H1.3 and H1.4 in facultative heterochromatin and their loss from actively transcribed regions [89,110]. However, of all the somatic subtypes, the affinity of H1.5 is perhaps the most contradictory when comparing different studies (Tables 2 and 3). This duality is seen *in vivo* as well. H1.5 synthesis begins early in S-phase, at a time when actively transcribed genes are being replicated [131] and phosphorylated H1.5 has been immunolocalized to mRNA processing sites [135,136]. H1.5 has also been localized to specific regions of gene *inactivation* in differentiating cells, indeed, showing specific distributions for different types of cells [137]. It interacts with protein Msx1 to specifically inhibit MyoD, a central regulator of skeletal muscle differentiation [138], *and* yet it activates von Willebrand factor when it binds a region of its promoter, as I discovered when I compared the partial sequence data provided by the authors of that study to the known H1.5 sequence [139]. The H1.5 interaction with DNA can be so specific it preferentially binds the Ω regulatory sequence in the mouse H3.2 gene, but has 100-fold lower affinity for the Ω sequence in the H3.3 gene despite the fact the two Ω sequences only differ by 4 out of 22 nucleotides [140]. Recent data suggests H1.5, like H1.4, interacts with SirT1 in regions of chromatin compaction [137]. Yet H1.5 is also the second major H1 targeted for poly-(ADP-ribosyl)ation (see the chromatographic trace in Fig. 1 of [83]) suggesting its greater accessibility to PARP than other subtypes except H1.2. H1.5 is now known to also create greater NRLs, in other words, greater linker space between nucleosomes, than the other subtypes when it is enriched on chromatin [66,141]. That would suggest H1.5 enriched regions of chromatin would have a more open conformation, a characteristic of actively transcribed genes.

None of this contradicts observations that H1.5 or H1.2, for that matter, are involved in both global and specific gene regulation. When binding to regions of more condensed chromatin, they contribute to the inactivation of gene transcription. In the case of H1.5, that could include interactions with SirT1 [137] and cooperative interactions with H1.4 to create heterochromatin, however, when binding in regions lacking subtypes H1.3 and H1.4, they establish a more open chromatin framework, one that allows access either for inhibitory proteins (MyoD) or transcription factors. A cooperative interaction with other H1 subtypes is not out of the realm of possibility as evidenced by the recent isolation of 107 binding proteins to H1°, one of which turned out to be H1.5 [59]. The binding occurs with the NTD and/or the GD of H1°, since the interaction was also isolated using an H1° CTD deletion mutant.

Given the intrinsic disorder of the CTDs, H1.5 could even have specific structural forms that interact with different proteins or specific nucleotide sequences depending on the circumstances of its surrounding environment. Its presence in a more open chromatin conformation would also explain the fact that H1.5 is the most phosphorylated subtype throughout the cell cycle [82], allowing it to be accessed by kinases. Thus, the ability to strongly condense chromatin in binding experiments and participate in the inhibition of specific genes does not contradict its dual nature. If H1.3 and H1.4 were to be knocked off the chromatin for some reason, H1.5 would still be bound to what is now a more open chromatin conformation and would still provide some level of compaction greater than that afforded by H1.2 alone. It is important to note that, as mentioned earlier, Raghuram et al. discovered hyperacetylation of the core histones does not uniformly disrupt H1 subtype binding to nucleosomes with H1.2 and H1.5 still binding hyperacetylated chromatin. In fact, H1.5 binding was “robust in spite of hyperacetylation” [99].

Fifteen years ago, it was conjectured that H1.5 populated a state of chromatin that was “poised” to be actively transcribed. Indeed, heat shocking fibroblast cells from a fetal cell line (GM02291) resulted in a loss of H1.5 on the heat shock protein 90α (Hsp90α) gene, leaving only the presence of H1.2 on a DNA sequence exhibiting a 22-fold increase in transcription within 3 hours after the stress stimulus [14] (Figure 3D). How this remodelling occurs has yet to be understood, but one can envision that simply removing the H1.5, either by further phosphorylation or some other post-translational modification, gets you to a state where only H1.2 is providing a minimal level of compaction and allowing for a high rate of transcription. Selective accessibility of a putative remodelling mechanism to the H1.5 located in open chromatin could explain the apparent specificity for the loss of this subtype from the active Hsp90α gene during heat shock while three Hsp90α pseudogenes continue to maintain a distribution of subtypes (H1.2, H1.5, H1.3 and H1.4) characteristic of inactive genes [14].

### 10.3. H1.3 (Parseghian’s H1^s^-2; Seyedin & Kistler’s H1d)

**Figure 4.**
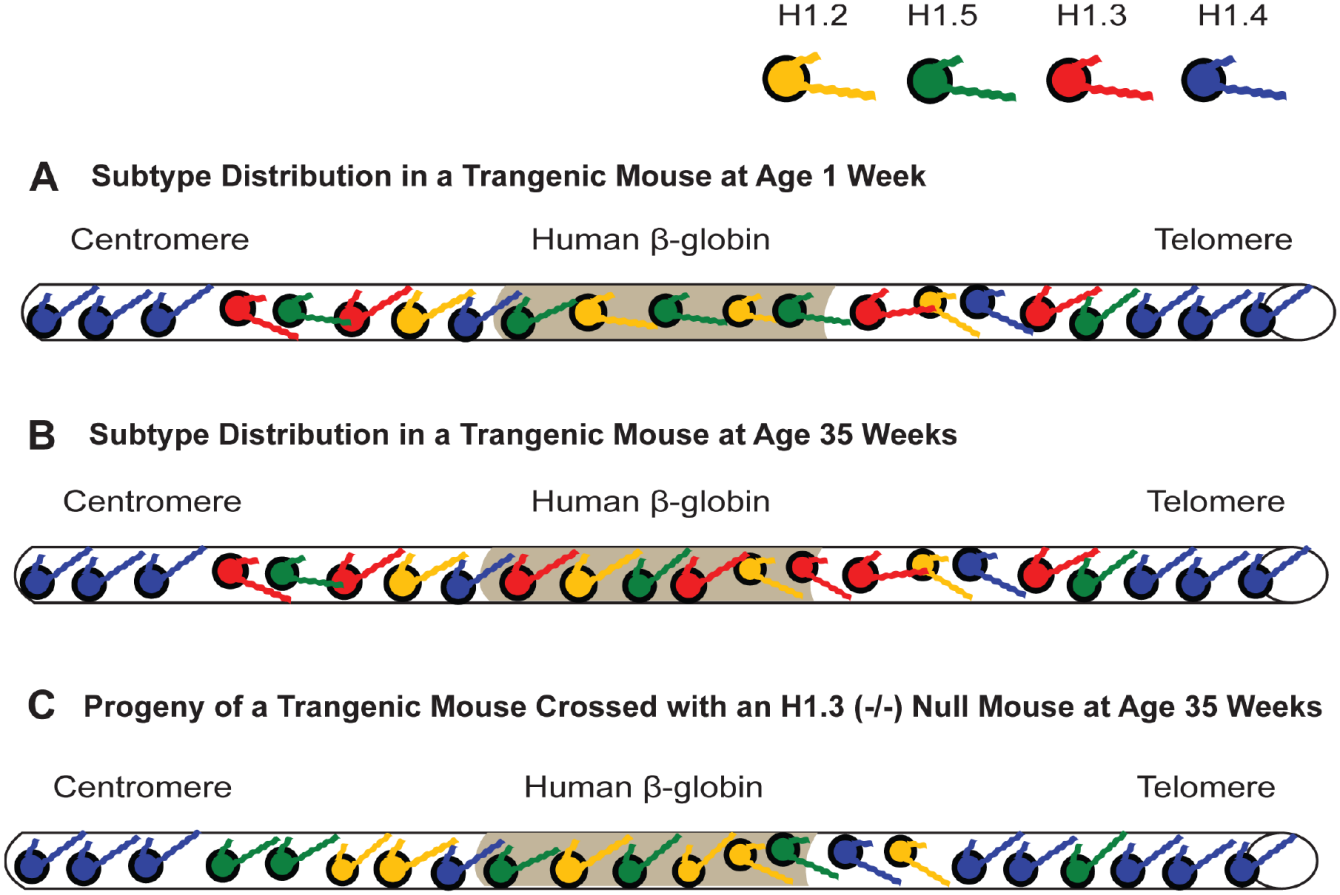
Subtype Distribution in a Transgenic Mouse. (A) A mouse carrying a human β-globin gene has an open chromatin conformation surrounding the locus and expresses the protein at 1 week after birth. (B) The same mouse at 35 weeks may have a diminished expression of the human β-globin gene due to the increased association of either H1.3 or H1.4, both of which promote silencing of the gene. (C) Crossing the transgenic mouse carrying a human β-globin gene with an H1.3 knockout mouse results in progeny exhibiting attenuation of the gene silencing at 35 weeks.

As suggested earlier, H1.3 is affiliated with gene inactivation, which is corroborated by its strong affinity and compaction of chromatin (Tables 2 and 3). Overexpression of a GFP-H1.3 fusion in human fibroblasts led to severe growth inhibition and a senescent phenotype [113]. The affiliation of H1.3 and H1.4 with chromatin compaction is strikingly demonstrated using knockout mice with hetero- and homozygous deletions of specific H1 subtypes. In a series of experiments, mice carrying a transgene of human β-globin in the center of a large acrocentric chromosome, far from the centromere and telomeres (Figure 4A), were crossed with knockout mice for each of the following H1 subtypes: H1°, H1.1, H1.2, H1.3 and H1.4 [6]. Expression of human β-globin is silenced gradually in red blood cells (RBCs) as the transgenic mice age (Figure 4B). However, in the progeny of transgenic mice crossed with homozygous knockouts of either H1.3 or H1.4, this silencing is significantly attenuated (p < 0.001) (Figure 4C). For example, at 35 weeks of age, >85% of RBCs in H1.4 (−/−) deficient mice and >70% in H1.3 (−/−) deficient mice expressed the human β-globin, whereas <40% of the RBCs expressed the same transgene at that age in control mice and homozygous knockout mice lacking both alleles of H1°, H1.1, and H1.2 [6]. The effect was dose dependent since mice heterozygous (−/+) for the H1.3 deletion still showed human β-globin expression in >40% of RBCs but not as many as the >70% seen with the homozygous H1.3 (−/−) knockouts. With some minor differences, these effects were seen for three different transgenes integrated at three different sites in the mouse genome.

**Figure 5.**
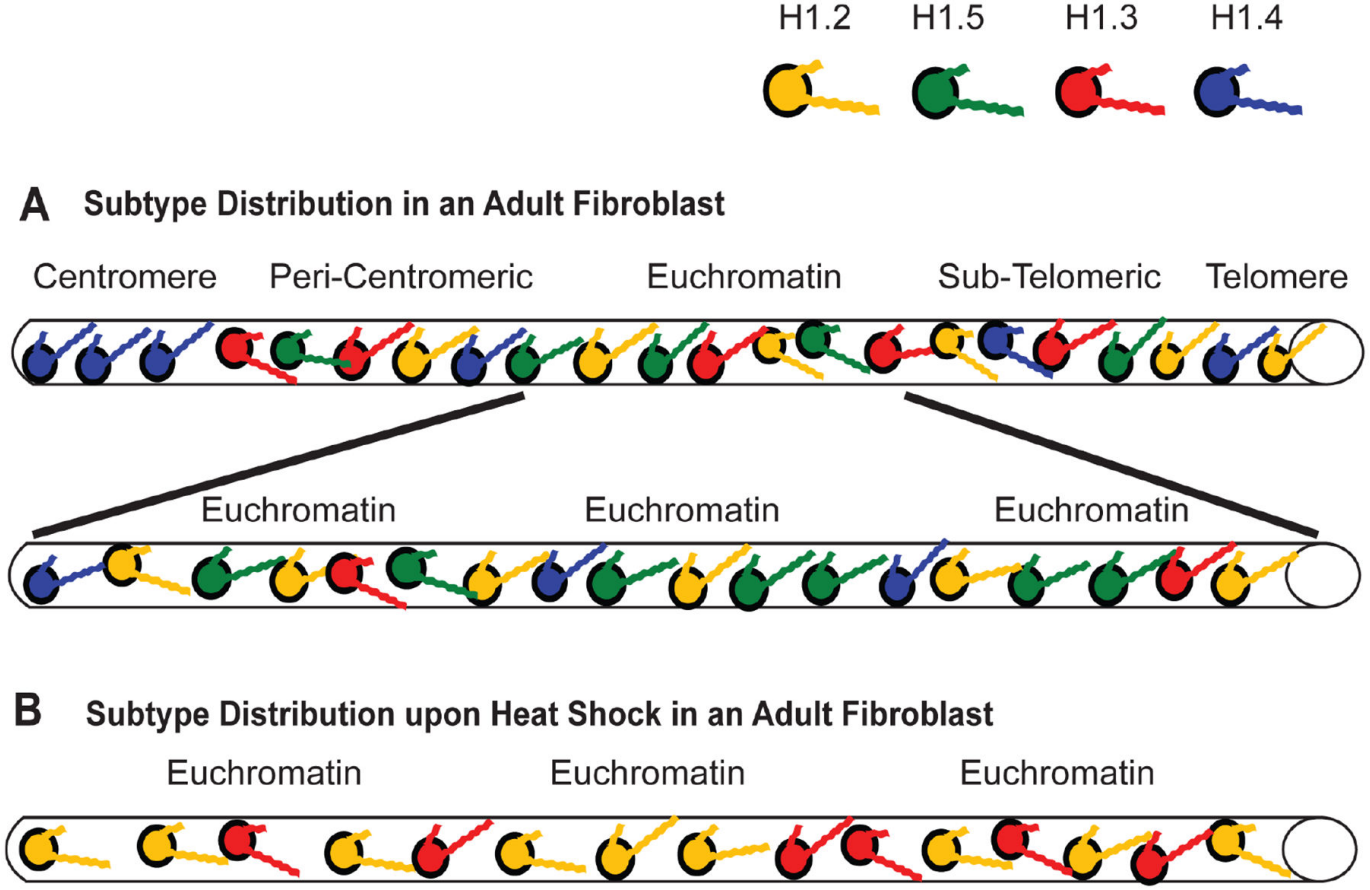
Subtype Distribution in an Adult Fibroblast Cell Line (GM1653). (A) Compared to fetal fibroblasts, adult fibroblasts have a greater association of H1.3 and H1.4 on actively transcribed euchromatin. (B) Heat Shock of GM1653 Cells. Actively transcribed genes upregulated during heat shock lose their association with H1.5 and H1.4, as is the case for fetal cells, however, H1.2 and H1.3 remain associated with the gene. Given the role H1.3 plays inhibiting transcription, this association correlates with attenuation of gene expression.

These observations further corroborate H1.3’s affiliation with gene inactivation as demonstrated in ChIP experiments using a fibroblast cell line (GM1653) derived from adult tissue [15]. Not only was there a greater presence of H1.3 on some *active* genes in the adult fibroblast cells compared to the same genes in a fetal fibroblast line, a clear increase in the presence of this subtype could be seen on the active Hsp90α gene as chromatin was harvested with increasing cell passage (Figure 5A; and see Figure 3 in [15]). Simultaneously there was no change in the distribution of H1 subtypes on the Hsp90α pseudogenes. Heat shock of the adult fibroblasts again left H1.2 affiliated with the induced Hsp90α, as well as the FGFR-3, genes. However, in these adult fibroblast cells, the H1.2 was accompanied by the increased presence of H1.3 (Figure 5B; and see Figure 4 in [15]). This correlates with the reduction in transcriptional induction seen in these cells during heat shock, as evidenced by the decrease in FGFR3 transcription comparing fetal and adult fibroblasts (see Figure 5 in [15]). When Izzo et al. (2013) compared the differential distribution of subtypes on a global scale in human IMR90 fibroblasts, they found enrichment of H1.3 with the X-chromosome compared to any of the other chromosomes investigated (Figure 1B in [90]), and they found it enriched in HP1 containing domains and depleted in domains with active transcription, more so than H1.4 or H1.5 (Figure 5B in [90]). To be fair, they also found this to be the case for H1.2, a finding that can be explained by H1.2 providing a basal level of compaction throughout the genome but unable to actually negatively impact transcription the way H1.3 does.

### 10.4. H1°

**Figure 6.**
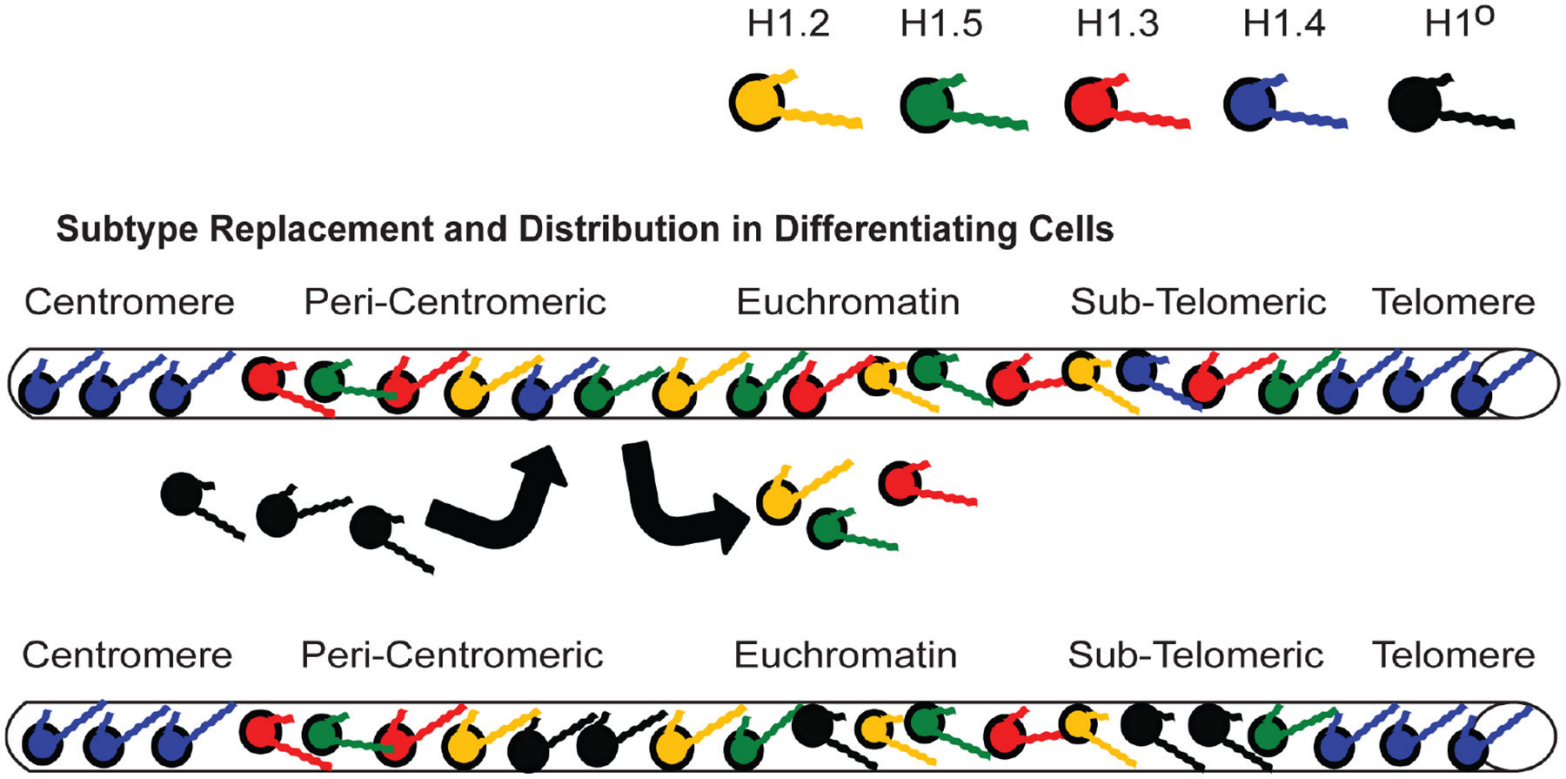
Subtype Replacement and Distribution in Differentiating Cells. Symbolized in black, H1° is a replication independent subtype that can replace replication dependent ones as they are catabolized in differentiated cells. Population of a chromatin segment with H1° generally leads to greater chromatin compaction and gene suppression, however, recent observations suggest some genes actually utilize the H1° to up-regulate expression.

Perhaps the most studied of the subtypes is H1°, whose early evolutionary divergence from the other subtypes [117] resulted in structural differences that allowed its full resolution and isolation using chromatographic and electrophoretic techniques. Unlike the other subtypes discussed so far, H1° has been referred to as a “replacement” or “differentiation specific” subtype and its high binding affinity and strong chromatin compaction characteristics (Tables 2 and 3) have only reinforced the belief that H1° is expressed in terminally differentiated cells in a replication independent manner [142] where it helps repress non-specific gene expression [143] (Figure 6). Surprisingly, H1° double knockout mice are viable and develop normally, with the loss of H1° compensated by the upregulation of H1.3 and H1.4 (see Figure 4 and Table 1 in [38]), two subtypes with sufficiently similar chromatin compaction characteristics (Table 3). As mentioned earlier, now there is evidence that, along with gene repression, there are specific genes whose expression is upregulated in the presence of H1° [112]. Replacing H1° with H1.3 or H1.4 may have very specific consequences. One possible example being the significant depletion of dendritic cells in H1° knockout mice [144]. So the role of H1° in gene regulation, a subtype we have been studying since the 1970s, is still not fully understood.

### 10.5. H1.1

**Figure 7.**
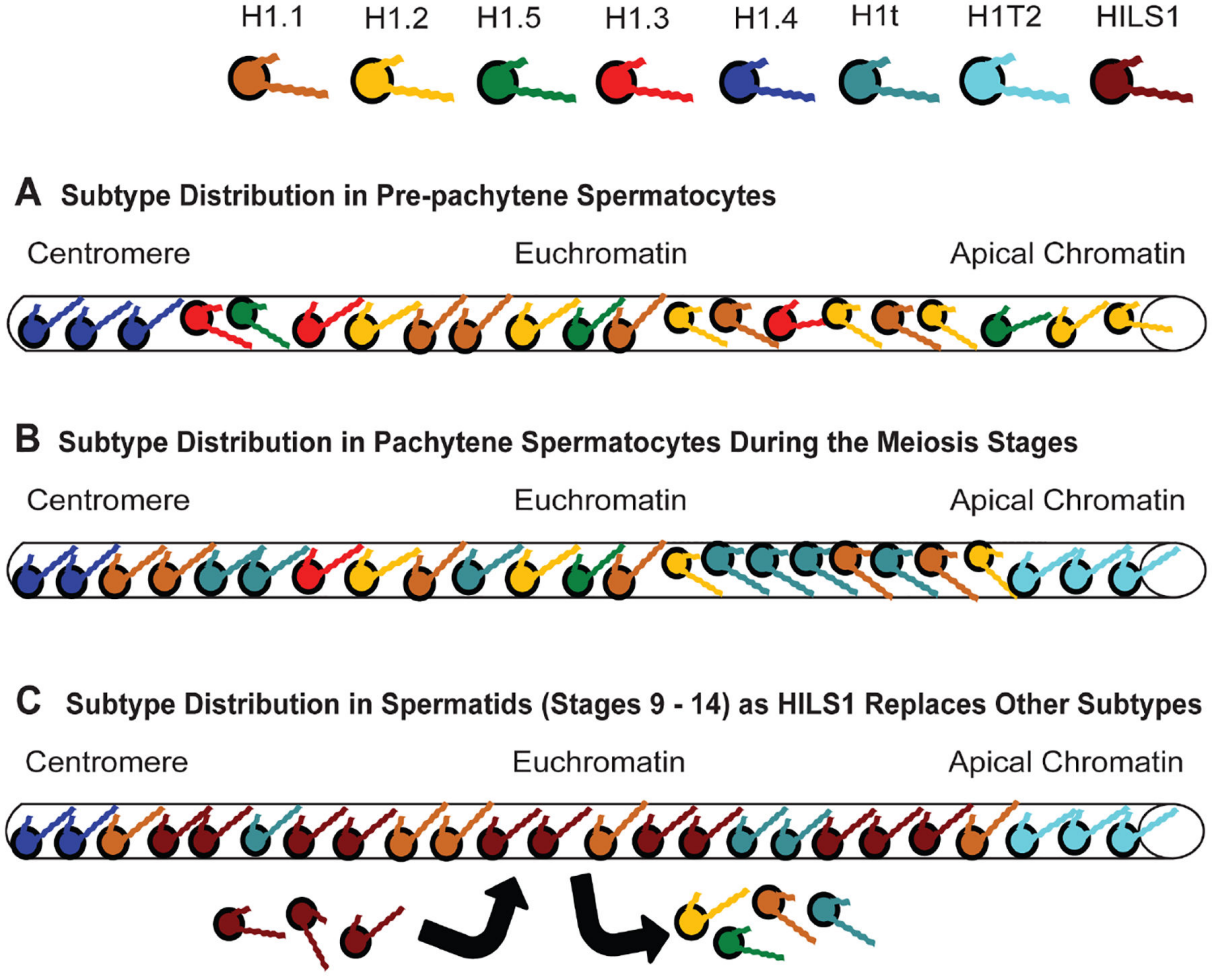
H1 Subtype Replacement During Spermatogenesis. (A) Pre-pachytene spermatocytes are populated with H1 subtypes with weak affinity for chromatin, including H1.1 (tan) and H1.2 (yellow). (B) Two other weak chromatin condensers, H1t (aqua) and H1T2 (light blue), are expressed during the pachytene stages of spermatogenesis, when cells are undergoing meiosis. H1T2 specifically localizes to a chromatin domain at the apical pole of the germ cell. (C) HILS1 (brown) replaces other subtypes during stages 9–14 in order to prepare the chromatin for further packaging into sperm.

On the other side of the chromatin condensation spectrum is H1.1, whose minimal compaction of nucleosomes is comparable to H1.2 (Table 3). H1.1 has been reported in several organs at birth with the subtype rapidly declining in amount as cells become differentiated, quiescent or both [145]. Transcriptional and immunological evidence demonstrates that soon after birth H1.1 expression is restricted to thymus, testis, spleen, lymphocytes and neuronal cells [146,147]. It has long been suspected of creating a more open conformation based on its continued presence in lymphocytes and pachytene spermatocytes (Figure 7A), cells that require minimal DNA compaction for somatic and meiotic recombination, respectively [148,149]. Corroboration comes from transgenic mice carrying a human β-globin gene that were crossed with H1.1 (−/−) knockouts. Loss of H1.1 expression in RBCs led to faster transgene silencing. Their progeny had a >50% decrease in transgene expression within 6 to 8 weeks after birth compared to control mice [6]. When an independent lab created H1.1 (−/−) knockouts, they found loss of the subtype did not affect spermatogenesis despite the subtype’s prevalence in these cells [149], however, they also showed the loss of H1.1 was offset by an increase in H1.2, H1.3 and H1.4 in testis compared to wildtype mice [150].

### 10.6. The Male Germline Subtypes (H1t, H1T2 and HILS1)

As pre-pachytene spermatocytes enter the pachytene phase with cells undergoing meiosis, there is an increased production of a germline specific subtype, H1t, reaching up to 35% of the H1 content in these cells until the stage of elongating spermatids [150] (Figure 7B) when HILS1 begins to replace the other H1 variants (Figure 7C) for eventual packaging of the sperm DNA using protamines [28]. Like H1.1, H1t null mutants (−/−) lacking both alleles develop normally and are fertile [39]. Although there is some microscopic evidence that H1t colocalizes with heterochromatin, this observation may be an artifact of an H1t antibody that does not detect phosphorylated forms of the protein in euchromatin [151]. The subtype is a weak aggregator of H1-depleted nucleosomes, weaker than the other subtypes, and is located in regions of the nucleus sensitive to DNAse I digestion ([107,152] and Table 3). H1t is also a poor inhibitor of DNA replication in a *Xenopus* egg extract system, unlike the other subtypes it was compared against (H1.2, H1.4 and H1°) [68]. In fact, it has been surmised that H1t in pachytene spermatocytes, along with H1.1, may facilitate a chromatin conformation amenable to meiotic recombination [149]. On the whole, H1t appears to be analogous to H1.2 in its prevalence throughout the spermatocyte genome, maintaining a basal level of compaction as H1.2 is diminished and proteins, like HILS1, begin to repackage the chromatin.

HILS1 is a germline specific subtype that helps transition the chromatin into the highly compacted state found in sperm. And while it aggregates polynucleosomes and binds mononucleosomes with a lower affinity than H1.1, it has a higher capacity to bind naked DNA than the latter subtype [28]. As discussed earlier with the case of H1.5, this dichotomy could be due to the intrinsic disorder built into the CTD of HILS1. It can still interact with nucleosomes *in vitro*, however, spermatid nuclei are largely lacking in core histones, so the HILS1 binds naked DNA and contributes to nuclear condensation using a different mechanism from what occurs in somatic nuclei. HILS1 is not only specific to spermatid nuclei, its expression is stage-specific, first being expressed in step 9 spermatids and decreasing abruptly in step 14 [28]. Despite retaining the tripartite structure of a globular domain with two tails possessing intrinsic disorder, this subtype has evolved a very limited function during a very specific phase in an organism’s development.

The last subtype to be discovered, H1T2, is specifically expressed in pachytene spermatids during spermiogenesis [29], along with H1t, H1.1, H1.2 and minor amounts of the other subtypes. Unlike the other subtypes, H1T2 specifically localizes to a chromatin domain at the apical pole of the male haploid germ cell after meiosis (Figures 7B, C), revealing a polarity between apical and caudal halves of the nucleus that is dependent on chromatin architectural proteins TRF2 and HMGB2 but is poorly understood [153]. The establishment of this polarity with H1T2 is essential for sperm development since deletion of the subtype in mice (H1T2 −/−) results in delayed chromatin condensation and aberrant nuclear elongation, in turn leading to severe morphological abnormalities, decreased sperm motility and loss of male fertility [29,154]. This makes H1T2 unique in more ways than one since null mutants of the other testis-specific H1 found in these spermatids, H1t, develop normally and maintain fertility [39]. While affinity of H1T2 to chromatin has yet to be reported, binding to DNA-cellulose columns reveals the subtype’s affinity is weaker than H1.2 HILS1 [154]. This is a more relevant observation than chromatin affinity anyway, since nucleosomes are largely missing from spermatid nuclei. As for interactions other than DNA, coprecipitation studies reveal H1T2 associates with protamines but does not interact either with H1.2 or HILS1, both prevalent in the pachytene spermatids [154].

### 10.7. The Female Germline Subtype (H1oo)

**Figure 8.**
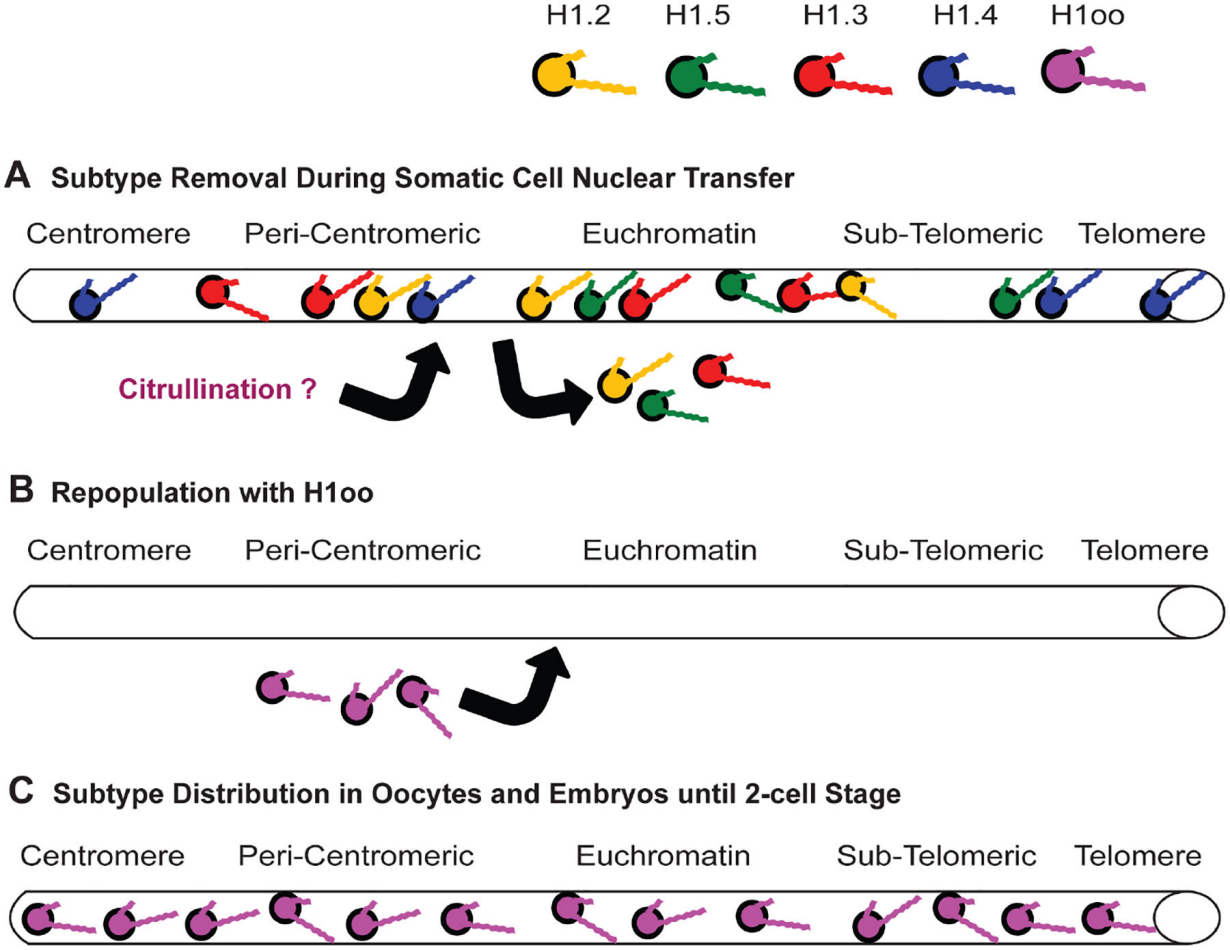
Replacement of Somatic H1 Subtypes in Oocytes. (A) Exogenous chromatin introduced into oocytes, either through sperm or somatic cell nuclear transfer, is replaced by H1oo (purple) in a two-step process. While the majority of sperm chromatin is packaged by protamines, some sperm chromatin still retains linker histones and nucleosomes. The linker histones are removed by an active mechanism in oocytes, possibly citrullination of a single arginine near the NTD and GD boundary. (B) The expanding chromatin is now available for binding by H1oo, and presumably core histones. (C) H1oo stabilizes the chromatin structure until the 2-cell stage of embryogenesis when somatic subtypes begin to repopulate the chromosomes.

The female counterpart to the male germline subtypes is H1oo; also stage-specific, it is relegated to the oocyte and the early embryo up to the 2-cell stage [93]. It exhibits a strong affinity for chromatin, when measured in terms of FRAP recovery times [57], yet poor chromatin condensation *in vivo* [155]. FRAP analysis from two independent labs found H1oo to have a 100–180 sec. recovery time and a t_1/2_ of ~ 30 sec. (Table 2), unfortunately, both had the GFP placed on the CTD rather than the NTD so interference in binding to chromatin may not reflect the true affinity values [57,95]. These relatively greater affinities compared with some other subtypes correlate with the localization of H1oo to the perinucleolar heterochromatin in germinal vesicle (GV) oocytes, providing a distinctive ring-like appearance to the nucleolus whether using H1oo antibodies or the GFP fusion protein [156]. Although a single copy gene, H1oo’s mRNA is alternately spliced such that two isoforms can be generated from the same gene: murine H1ooα is 304 amino acids long and has a net positive charge of 50 from its amino acid composition, whereas the CTD truncated H1ooβ is only 246 amino acids long and has a net positive charge of 43 [156]. Both isoforms have similar localizations to the perinucleolar heterochromatin in GV oocytes so a functional difference has yet to be determined [156], however, if correlation of H1 lengths to chromatin affinity holds true, the H1ooβ should have a weaker affinity and perhaps create a more open chromatin conformation than the H1ooα. H1oo’s chief characteristic is its ability to rapidly, within 5 min., occupy exogenous chromatin introduced into the egg whether it arrives on the head of a sperm or by injection of a somatic nucleus [93]. Even in the protamine packaged sperm nucleus, ~ 15% of the chromatin is still organized by histones [157], including H1s [158], in stretches of DNA that can be up 50,000 bp [157]. This exogenous chromatin is stripped of any residual H1s possibly by the citrullination of a single arginine site (R-54) near the NTD–GD boundary [105] (Figures 8A). H1oo then populates the decondensing nucleus (Figure 8B, C). Unlike its residence in the periphery of the oocytic nucleolus, there is evidence that it binds throughout the exogenous chromatin [93] where, based on *in vitro* reconstituted nucleosome models, it establishes an environment amenable to chromatin remodelling agents for commencement of the organism’s development programs [155].

### 10.8. H1x

**Figure 9.**
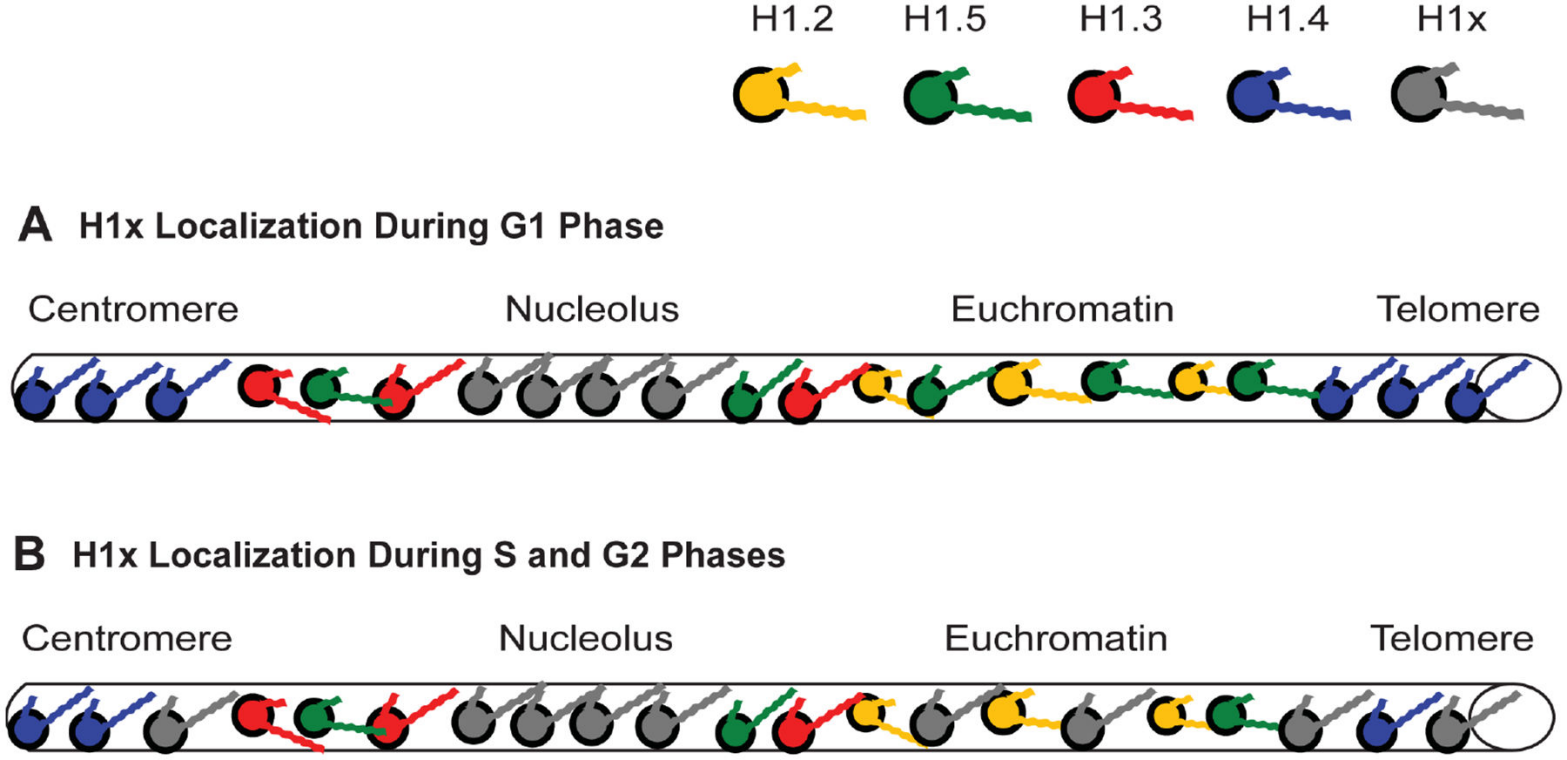
H1x Localization. (A) H1x (gray) is largely located in the nucleolus during the G1 phase of the cell cycle. (B) It redistributes throughout the nucleus during the S and G2 phases.

The role of H1x may be unique among the subtypes with its cell cycle-dependent translocations during the stages of interphase. It localizes to the nucleolus during G1 (Figure 9A), then it appears to evenly distribute throughout the nucleus during the S- and G2-phases (Figure 9B) [159]. These studies were conducted in HeLa cells and in SV-40 transformed fibroblasts (SV-80 cells). When an independent group studied H1x in a human breast cancer line, the results were different. H1x was not in the nucleolus, rather it was associated with actively transcribed regions of chromatin, including constitutively expressed genes, RNA polymerase II-enriched regions, hypomethylated CpG islands and the 3’ end of expressed genes [160]. This latter finding may better reflect the distribution of H1x during the S- and G2-phases. During mitosis, H1x is located on the chromosomes, particularly at the chromosome periphery [103]. Chromatin compaction studies in a low salt buffer (20 mM KCl) [66] and micrococcal nuclease digestion of chromatin from tissue cultured cells [161] both suggest H1x is enriched in condensed chromatin during the G1 phase. This is supported by an immunohistologic analysis that localizes the subtype to the granular component in nucleoli, a region consisting of condensed chromatin and devoid of RNA polymerase I-driven transcription [159]. And yet, efficacy determinations using FRAP [103] and nucleosome repeat length [66] analysis suggest H1x has a weak affinity for chromatin (Tables 2 and 3). This could explain its distribution seen in the human breast cancer line and its relative mobility throughout the nucleoplasm at S and G2. The mechanism of H1x’s translocation from the nucleoli into other subnuclear compartments is not known, perhaps a post-translational modification that disrupts the CTD binding to nucleolar sequences, causing the intrinsically disordered protein to interact with a wider array of DNA throughout the nucleus. One purpose for H1x’s migration may be wholly unique for an H1 subtype: as a cell enters mitosis, H1x has a role in chromosome alignment and segregation. RNA interference studies targeting H1x detected an 8-fold increase in misaligned (1–10 chromosomes not aligned at the metaphase plate) and 32.5-fold increase in non-aligned (>10 chromosomes not aligned at the metaphase plate) chromosomes [103].

## 11. Applying This Model to Explain the H1 Knockout Data

**Figure 10.**
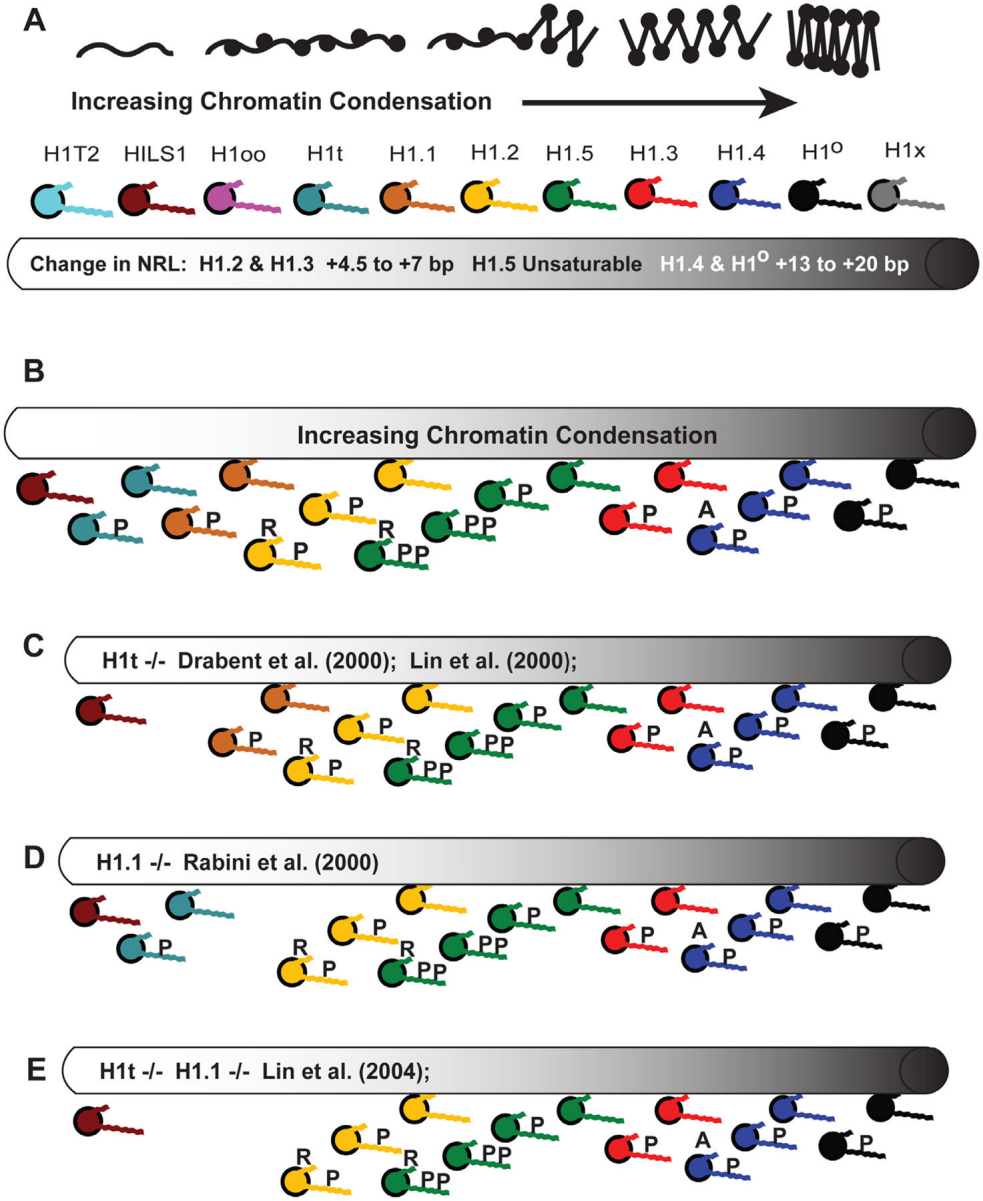

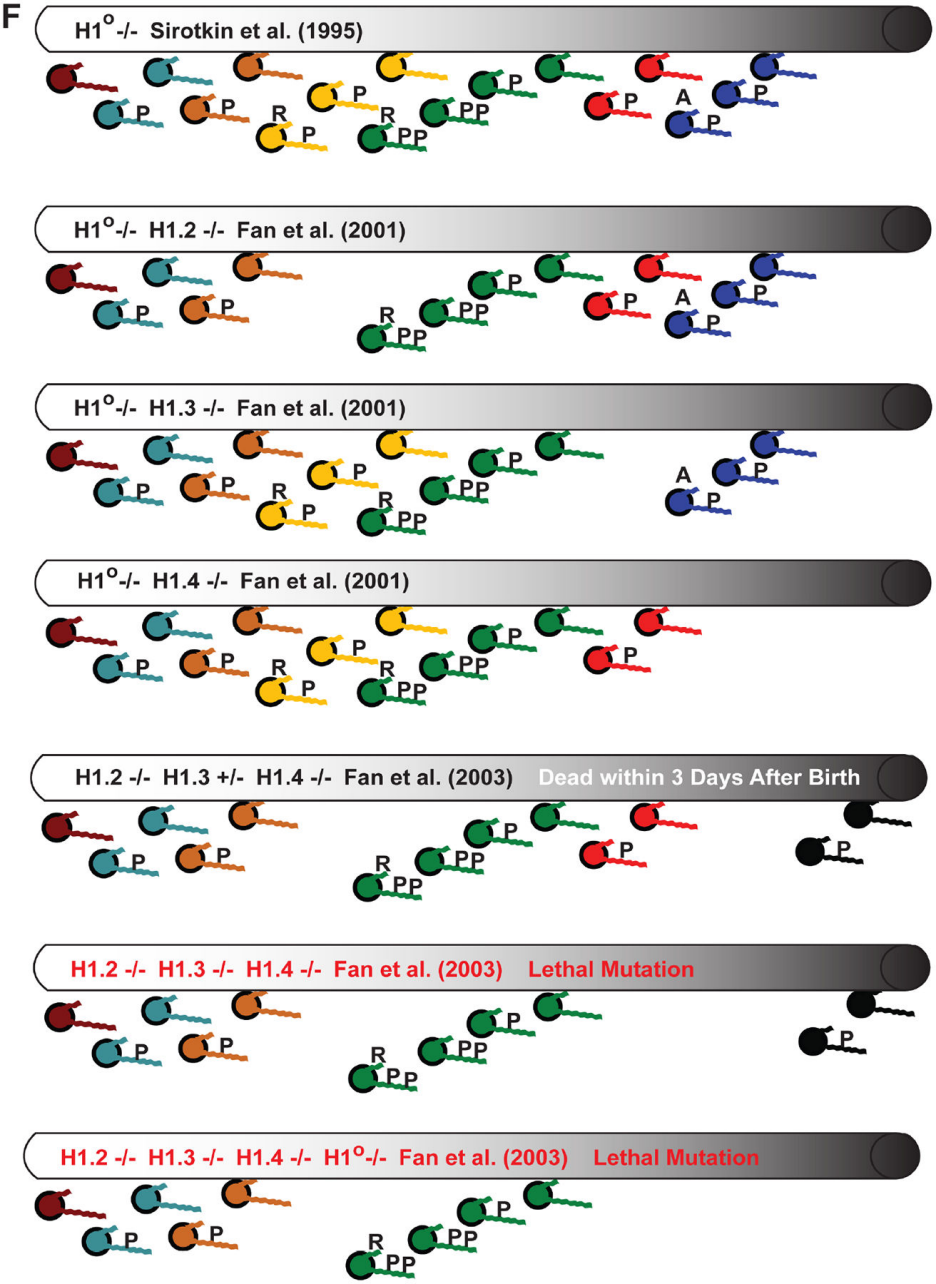
A Working Model of H1 Subtype Functionality. (A) Subtypes are aligned from left to right according to their affinity and compaction of chromatin. These are estimates that may change, particularly in the case of H1x as further investigations clarify its role in the nucleus. Diversity of H1 subtype interaction with chromatin also results in a diversity of NRLs. Both affect chromatin structure as illustrated above with 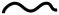 representing DNA and ● representing nucleosomes. (B) We must view the diversity of H1 subtypes as a continuum of molecules that can create varying levels of compaction, some of which provide redundancy in the system. Some post-translational modifications are symbolized in the model, although this is not meant to be a comprehensive survey of all the modifications occuring to H1s. Phosphorylation (P), Poly(ADP-ribosyl)ation (R) and Acetylation (A) are modifications that result in more open chromatin conformations, hence, the positioning of modified variants away from the chromosome and to the left of their respective unmodified subtype. (C–F) Using the model to illustrate the range of chromatin stabilization still available in H1 subtype knockouts described in the literature. The publication describing each knockout is listed above each illustration.

If we were to view multi-cellular lifeforms as engineering projects and the histone proteins as instruments for regulating access to the genomic blueprints, then the H1 family of histones provides a diverse array of tools that are adjustable for any level of transcriptional control (Figure 10A). Each subtype can be versatile in its interaction with DNA and other proteins thanks to the intrinsic disorder built into the structure. When combined with different degrees of chromatin compaction afforded by differential phosphorylation, poly-(ADP-ribosyl)ation, acetylation and other modifications of the subtypes, a continuum of chromatin states emerges allowing for fine regulatory control (Figure 10B). Just as importantly, this continuum would have some overlap allowing cells to compensate in the event a subtype or two were lost to mutations caused by nature or deliberate knockout caused by a curious scientist.

The creation of knockout mice missing one or more subtypes has helped bring the adaptability of the H1 variants into focus. Some of the earlier studies knocked out subtypes with weak chromatin aggregation capabilities, such as H1t, and found no effect on viability or fertility [39,162]. Two separate groups created H1t knockout mice and studied the effects on pachytene spermatids and spermatogenesis (Figure 10C). The apparent lack of any detrimental phenotypes in H1t (−/−) mice had one group suggesting the remaining subtypes compensate for the loss of H1t in order to maintain the same H1/nucleosome ratio found in wildtype mice [39]. The three prevalent subtypes in pachytene spermatids are H1t, H1.1 and H1.2, with H1.1 accounting for 50% of the total H1s [39], so a loss of H1t in a particular region of chromatin would be likely compensated for by H1.1, another weak chromatin condenser. The other group of researchers did not find H1.1 or H1.2 replacing the lost H1t on chromatin and surmised the H1t provides such a low level of compaction, its loss did not affect the open chromatin structure that it maintains [163]. Placing the subtypes in an increasing order of relative chromatin condensation capabilities allows us to now see why some of these studies provided the surprising answers they did (Figure 10A,B). H1t is relegated to a specific cell line for a very specific period of time. It’s removal was not consequential to the overall maintenance of pachytene chromatin given the prevalence of other open chromatin subtypes (H1.1, H1.2) and their post-translationally modified isoforms throughout the genome. Knocking out H1.1 (Figure 10D) similarly had no effect on spermatogenesis or fertility and appears to show an increase in H1.2 to compensate the loss of H1.1 [150]. Removing both H1t and H1.1 from the development program begins to have some impact on specific genes (Figure 10E), however, the mice were fertile, exhibited homologous recombination, repaired double strand breaks, condensed their chromosomes in a meiotic metaphase I configuration and produced sperm comparable to wildtype mice [164]. The authors note a 25% decrease in the H1/nucleosome ratio in the progeny may be causing a *decrease* in the expression of 17 genes (out of 9,000 screened by microarrray analysis), however, a careful review of the data finds a significant increase in subtypes H1.3 and H1.4 (subtypes with greater condensation characteristics) and a decrease in the remaining “open conformation” subtype, H1.2 (see Table II in [164]). Redistribution of some H1.3 or H1.4 into regions formerly occupied by H1t or H1.1 could account for the decreased expression of specific genes.

To reiterate, we are not discounting the possibility that subtypes can have broad interactions with chromatin and, through the process of intrinsic disorder, still interact with specific genes probably with the assistance of other protein co-factors. In fact, it is very likely this is the case given observations of specific subtype and gene interactions (e.g. [138,140]) and the fact that none of the 11 subtypes in mammalian cells have been lost in 400 million years of evolution. However, on a broader scale, we might want to consider genome organization as a balancing act between subtypes that condense chromatin more efficiently and subtypes whose role it is to prevent encroachment of these variants into regions of active transcription. The concept goes both ways, as the loss of efficient chromatin condensing subtypes can cause havoc by preventing stabilization of heterochromatin. Such a concept does not contradict what has been learned about the actions of heterochromatinization proteins, such as the Sir proteins, it simply provides another level of global contol that complements it.

Thanks to the work of the Skoultchi lab, we can look at stepwise removal of subtypes and see how far this continuum can be disrupted before development is fatally affected (Figure 10F). When viewed in relation to the overall subtype composition, knocking out H1° does not disrupt development of a viable, fertile mouse [38], even though it effects specific physiological systems, such as dendritic cells [144]. One can argue that in the real world, outside the carefully controlled environment of the laboratory, such a defect could be quite fatal, hence, the loss of H1° is not evolutionarily advantagous in nature. A review of the data finds the loss of H1° compensated by increases in H1.3 and H1.4, both strong binding subtypes, like H1° (see Figure 4 in [38]). Double knockouts of H1° with H1.2, H1.3 or H1.4, still allowed for other subtypes and their post-translationally modified isoforms to maintain a functional nucleus [40] (Figure 10F). One can only speculate at this time if the loss of H1.4 did not disrupt SirT1 guided heterochromatinization because H1.5 replaced H1.4 in the process. Recall H1.5 is associated with SirT1 in heterochromatic regions [137]. It should be noted, as far as this author is aware, creation of an H1.5 deletion mutant has not been reported. Triple knockouts of H1.2, H1.3 and H1.4 proved embryonically lethal with none found beyond stage E11.5 [5]. Embryos analyzed from this genotype showed multiple developmental defects. Given the deletion of H1.3 and H1.4, these embryos also showed an increase in H1°, however, compensation mechanisms using this strong chromatin condenser were insufficient to replace the loss of both subtypes [5]. Strikingly, leaving a single wildtype allele of H1.3 was sufficient for rescuing the embryonic lethality, but this combination (H1.3 +/−, H1.2 −/−, H1.4 −/−) did not provide sufficient subtype variation to regulate the genome and allow mice to survive beyond 3 days. Knocking out H1° to create a quadruple knockout of it and H1.2, H1.3 and H1.4 also resulted in an embryonic lethal mutant—a forgone conclusion in view of our model [5] (Figure 10F). An important product from this work was the isolation of embryonic stem cells (ESCs) from triple homozygous null mutants (H1.3 −/−, H1.2 −/−, H1.4 −/−) during stages E7.5-E9.5 which have provided a very useful tool in understanding the link between H1 functionality and multicellular development [165].

## 12. Applying This Model to Understand NRL Data

Normally, ESCs can be differentiated into numerous cell types, however, these triple knockout (KO) cells tend to retain gene expression characteristic of undifferentiated cells [166]. Their embryoid bodies lack structures representing the three germ layers. Furthermore, these ESCs are deficient in undergoing neural differentiation when exposed to all-trans retinoic acid, and resistant to spontaneous differentiation upon removal of leukemia inhibitory factor. This harkens back to our earlier suggestion that multicellular organisms with diverse cell types have a greater number of linker histone subtypes (Table 4). Researchers studying this particular ESC line draw the conclusion that chromatin compaction may mediate pluripotent stem cell differentiation and changes to the condensation patterns disrupts silencing of key pluripotency genes by impairing DNA methylation of those genes. The researchers determine that the loss of H1.2, H1.3 and H1.4 represents a 50% loss of total H1 compared to wildtype mouse cells. This deficit changes the overall H1/nucleosome ratio from 0.46 found in wildtype ESCs down to 0.25 [165], a ratio they reason causes the global decrease of the NRL from 189 bp down to 174 bp, in turn leading to greater nucleosome coverage of the chromatin to compensate for the lost charge neutralization usually provided by the H1 linker histones. They also note a decrease in the NRL from 183 to 173 bp for a housekeeping gene, so the shift in length is not solely relegated to inactive or heterochromatin [165]. While it is reasonable to think there is only so much linker histone you can delete before affecting proper differential condensation of the chromatin during an organism’s development, we now know that only loss of the ground state subtype, H1.2, can cause global shifts in nucleosome repeat length identical to those reported for the triple KO ESCs. Knocking out H1.2 using shRNA resulted in a reduction of the global NRL in a human breast cancer line from 184.7 to 173.5 bp, as has already been noted [7]. H1.2 represents only about 20% of the H1 content in these cells. Depleting subtypes H1.4 or H1.5, which have similar or higher percentages of total H1, had no such effect. Only transfection of the cells with shRNA resistant H1.2, and not the other subtypes, restored NRL to 184.7 bp [7].

The influence of H1.2 and the other subtypes on chromatin structure are mediated by their affinity for DNA and may be mediated by their interaction with other proteins, however, nucleosome repeat length is emerging as the key mechanism directing chromatin condensation [9] with different NRLs generating different levels of compaction [133]. The change in NRLs caused by each subtype when expressed in *Xenopus* oocytes, as reported by Oberg et al. (2012), are included in Figure 10A to illustrate that loss of subtype variation can also mean loss of NRL variation throughout the nucleus [141]. Because these measurments were taken by expressing individual subtypes in *Xenopus* oocytes, there is a risk that the NRL values may be influenced by the presence of the amphibian equivalent of H1oo (known as B4) in the chromatin. These same issues arise when expressing any subtype within a chromatin matrix already possessing H1s. For example, Cao et al. (2013) use the triple KO ESCs and wildtype ESCs to knock-in tagged versions of H1.2 and H1.3 and report finding localization of both subtypes in major satellite DNA and their overall distribution in the genome to be quite similar [91]. They also indicate addition of these tagged versions of H1.2 and H1.3 may increase the NRL at major satellite DNA by ~ 13 bp, a larger value than reported by Oberg et al. (2012) for these two subtypes (Figure 10A). The differences in results could be due to the fact that oocytes and ESCs already have their own very different complements of H1 subtypes being expressed and exogenous H1s introduced into the nucleus have modified interactions with the chromatin that do not accurately reflect true values of NRL spacing or nuclear localization for the subtype under study. The recent publication of a mathematical model that computes the optimal length of the histone bound DNA based on energy minimization and the electrostatic charge of linker histones [167], provides biophysicists with the opportunity of estimating which NRL values are theoretically possible with each of the H1 subtypes. The model is already in agreement with experimental NRL data [168]. Therefore, based on such a theoretical analysis, researchers can then put the NRL results reported by other groups in better context. If experimental values for each subtype differ significantly from the calculated values, one can begin to search for other factors influencing the NRL, such as post-translational modifications of the H1s.

The triple KO ESCs do highlight the versatility of the H1.5 subtype since it must largely organize the different states of euchromatin in these cells; assuming the increased expression of H1° helps organize the heterochromatin [5]. Fan et al. (2005) visually classified polynucleosomes (n = 20–40 nucleosomes) isolated from wildtype and the triple KO ESCs into 4 categories according to chromatin conformation (see Figure 3 in [165]). There was a significant increase in the beads-on-a-string conformation with visible linker DNA and a decrease in the condensed chromatin 30 nm fiber conformation for the triple KO ESCs compared to wildtype ESCs. Given the observation from independent labs that increasing concentrations of H1.5 create longer NRLs than subtypes [66,141], combined with the observation longer NRLs (197 bp) form 30 nm fibers and short NRLs (167 bp) form thinner, topologically different fibers [133], it is surprising to find significantly fewer polynucleosomes with the 30 nm fiber conformation in ESC lines that only have H1.5 and H1° as their major subtypes. Since others have reported the localization of these two subtypes with condensed chromatin, one might have thought there would be only two conformations among the polynucleosomes isolated from the triple KO ESCs: either densely compacted polynucleosomes containing the subtypes or beads-on-a-string without any H1. However, Fan et al. (2005) noted 4 categories, including hybrid polynucleosomes with more than one conformation, in other words, beads-on-a-string and more condensed chromatin, co-existing on 20–40 nucleosome structures (see Figure 3 in [165]). Why would these exist at all since one would expect with the lack of H1s this heterogeneity of compaction on these hybrid polynucleosomes would be obliterated with nucleosome repositioning using any one of several dynamic processes (reviewed in [169])? On the other hand, with the observation that H1.5 interacts with SirT1 [137] shouldn’t heterochromatinization make it unlikely one would isolate a hybrid polynucleosome? We can explain these hybrid polynucleosomes by envisioning H1.5 as a subtype whose interactions with DNA are governed in the context of the surrounding chromatin. The same H1.5 can act as a condenser in cooperative interactions with other proteins (SirT1, H1°) and still interact with more open chromatin when it is phosphorylated [82] or poly(ADP-ribosyl)ated [83] even when in close proximity to heterochromatin. The presence of H1.5 in the less condensed chromatin can inhibit nucleosome repositioning and given the fact that H1.5 is the most phosphorylated subtype throughout the cell cycle [82], a post-translationally modified H1.5 allows for other proteins, such as transcription factors, to access the DNA. In this way, hybrid polynucleosomes found in these ESCs may illustrate how H1.5 can provide sufficient variation in chromatin compaction to help embryonic development up to stage E11.5 for the triple KO mice.

## 13. This is the End: Applying This Model to Understand the Aging Telomere

**Figure 11.**
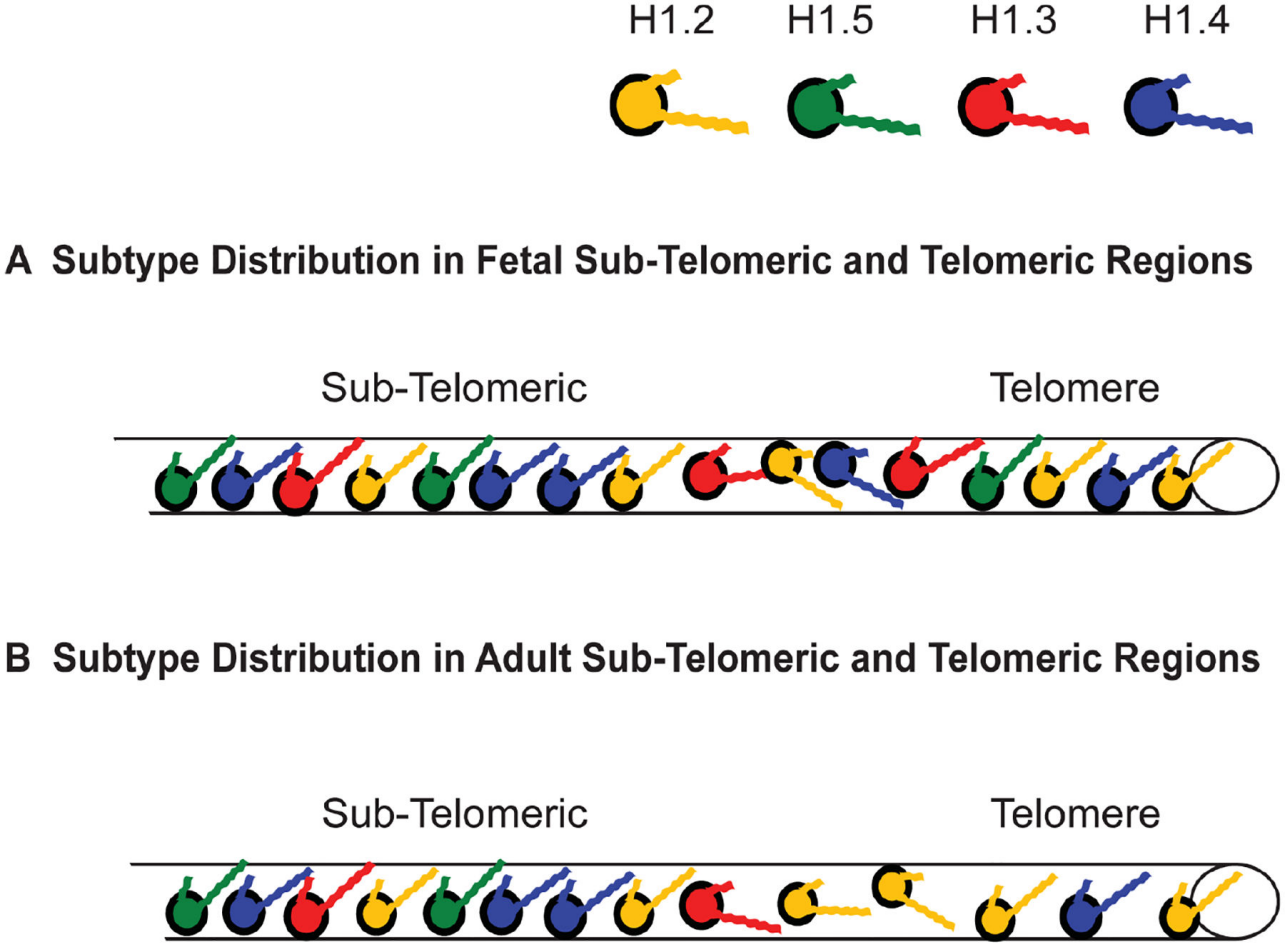
Telomeric Heterochromatin Organization. (A) Telomeric heterochromatin has a diverse set of H1 subtypes in fetal tissues. (B) This diversity is reduced during aging where a single subtype, H1.2 (yellow) predominates upon a region of heterochromatin characterized by loss of nucleosomes and total H1. Subtelomeric repeat sequences (3.3 kb repeat sequence) continue to maintain a heterochromatic distribution of H1 subtypes in aging cells, so there is a yet to be characterized barrier between telomeric and sub-telomeric heterochromatin.

It is appropriate to close this discussion by commenting on the telomeric ends of chromosomes. The relative total amounts of H1 associated with telomeric sequences is less than other heterochromatic regions and is particularly lower in adult human fibroblasts, where ~ 70% of the H1 content is made up of H1.2 [15]. The presence of a more homogenous H1 content on telomeres in aging cells is consistent with sharper NRL banding patterns seen in 3–4 month old rat liver nuclei [170]. The reduced H1 to nucleosome ratios on aging telomeres compared to bulk chromatin appear to be universal since they are corroborated by similar observations in a plant [171]. If all H1 subtypes had similar interactions with chromatin, telomeres in aging cells should have an even distribution of the subtypes in the same way they do in fetal cells, albeit at a lower H1 to nucleosome ratio. This is clearly not the case. It appears there is a selective depletion of the other subtypes leaving only H1.2 residually binding to telomeric heterochromatin and affecting the compaction of this subnuclear region (Figure 11).

The aging mechanisms bringing about this circumstance are still being clarified, however, some of the key drivers are now understood. As cells age, they must combat a number of stress inducing stimuli, including telomere shortening. This form of genomic stress, in the form of chronic exposure to DNA damage response (DDR) signals from the telomeres will eventually trigger a series of events leading to cell-cycle arrest that is commonly referred to as replicative senescence (for a good review see [172]). One of the DNA damage responses involves SirT1, which deacetylates H1.4 (at lysine-26) to promote DNA repair at a damaged locus [173]. During aging, SirT1 increasingly remobilizes from heterochromatic and transcriptionally inactive regions, where it represses gene expression, to regions of DNA damage. This constant remobilization, combined with a decline in SirT1 expression in aging mice, leads to the inadvertant deregulation of genes normally repressed in younger cells [173]. A downward spiral is established in which loss of SirT1 control over regions that should remain transcriptionally inactive further promotes the aging process. It is surmised this includes expression of genes that inhibit histone translation. Comparisons of histone biosynthesis in synchronized early and late-passage human diploid fibroblasts found H3 and H4 expression decreases with aging (>40% reduction), whereas, *H1 expression is not down-regulated* [174]. Surprisingly, aging can be partly reversed and longevity increased by the ectopic expression of core histones [175].

So how does the proposed model in this paper help us understand the unique chromatin structure in the telomeres of aging cells? In fetal fibroblasts, the distribution of H1 subtypes in telomeres is similar to that found in subtelomeric repeat sequences (the 3.3 kb tandem repeat [176]) and in other inactive chromatin (Figure 11A). Each of the major somatic H1 subtypes can be found associating with the telomeric chromatin. With aging, the reduction in H3 and H4 production results in reduced nucleosome occupancy of telomeres, while the loss of SirT1 would result in the hyperacetylation of H1.4 [86] and a lower binding affinity leading to its eventual migration from heterochromatin, particularly telomeric regions where there are fewer nucleosomes to bind. Since SirT1 also associates with H1.5 in heterochromatic regions, the same may occur with this subtype [137]. This loss of SirT1 deacetylation activity and increasing access of kinases to the H1 NTD and CTD should reduce the binding affinity of the subtypes and could explain the selective depletion of some H1 subtypes from the aging telomeres. Unlike the other somatic subtypes, H1.2 is replication independent [132] and its continued expression would result in its eventual presence as the major subtype, maintaining a minimal level of compaction on the telomeres (Figure 11B). This minimal condensation could result in the fewer nucleosomes present in some sections to reposition closer together in order to compensate for the lost charge neutralization, which could explain the smaller NRLs observed on telomeres from 3–9 month old rat liver cells [177] despite the presence of H1.2, which under normal circumstances maintains a 184.7 bp NRL. The more open chromatin conformation provided by H1.2 helps explain why telomeres in aging fibroblasts replicate much earlier in S-phase than other heterochromatin still in a condensed conformation [174]. And finally, the model helps explain the evolutionary advantage of having H1.2 enrichment of aging telomeres. H1.2 is a major target for poly(ADP-ribosyl)ation [83,128,129], a post-translational modification closely associated with generating an open chromatin conformation at DNA strand breaks for access of repair mechanisms [85], such as the ones activated from the DDR signals caused by telomere shortening.

## 14. Conclusion

I hope the model described here will achieve the following: 1) provide some clarity to the decades of observations made by colleagues, 2) harmonize those observations by assigning the subtypes to a single nomenclature so that the reader does not have to go back and piece it altogether themselves, and 3) establish a working model that puts all of this data into context and allows for a global understanding of H1 subtypes and their functions. As I reiterated at the start, this review is a companion to its predecessor, which covered much of the H1 work from the 1960s through the 1990s and first proposed the model of subtype functionality detailed here [2]. As discussed, recent observations using innovative new methods have lent further credence to the original proposal. It is my hope this proposal sparks discussions, further inquiries and, most importantly, serves as an adaptable model as new data further refines our understanding of H1 subtype functionality.

## Figures and Tables

**Table 1.** Correlation of nomenclatures.

Somatic Subtypes				
Human Official Symbol	Doenecke [18]	Parseghian [30]	Seyedin & Kistler [23]	Comments
HIST1H1A	H1.1	H1a	H1a	Replication Dependent [142], Synthesis starts in early S-phase [131]
HIST1H1B	H1.5	H1^S^-3	H1b	Replication Dependent [142], Synthesis starts in early S-phase [131]
HIST1H1C	H1.2	H1^S^-1	H1c	Replication **In**dependent [132]; Makes two mRNAs, one has polyA^+^ tail [132]
HIST1H1D	H1.3	H1^S^-2	H1d	Replication Dependent [142], Synthesis starts in early S-phase [131]
HIST1H1E	H1.4	H1^S^-4	H1e	Replication Dependent [142], Synthesis starts in **mid-**S-phase [131]
H1F0	H1°	H1°	H1°	Replication **In**dependent [142], Found in terminally differentiating cells [178]
H1FX	H1x	Localized to nucleolus in interphase and required for chromosome alignment and segregation during mitosis [103], Replication **In**dependent [26,161]		

Germline Subtypes			
Human Official Symbol	Original Designation	Tissue Specificity	Comments
HIST1H1T	H1t	Testis specific	First isolated by [25], Replication Dependent [179]
H1FNT	H1T2	Spermatid specific	First isolated by [29], Replication **In**dependent [43]
HILS1	HILS1	Sperm specific	First isolated by [28], Replication **In**dependent [43]
H1FOO	H1oo	Oocyte specific	First isolated by [27], Replication **In**dependent [43]

**Table 2.** FRAP Characteristics of H1 Subtypes.

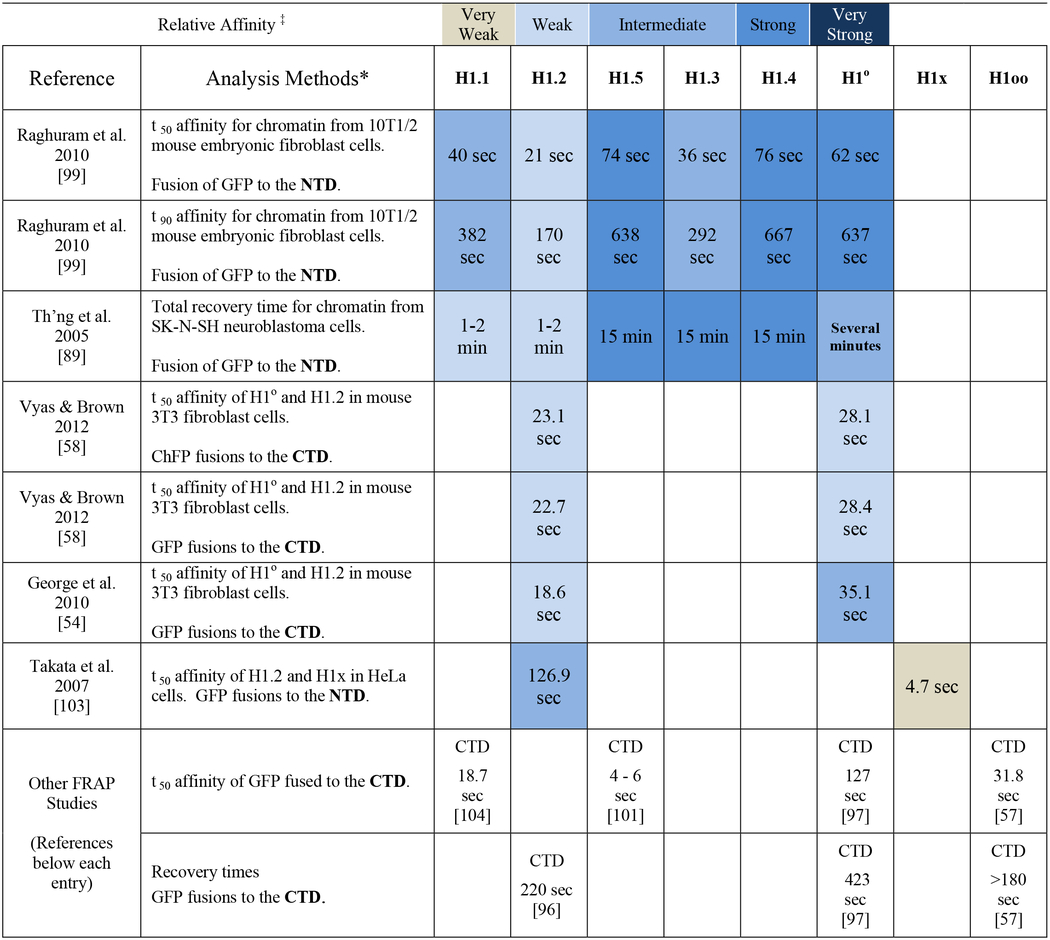

‡ Most of these studies use different cell lines. To harmonize findings from different cell systems, I have established a color code to visualize the level of affinity for each subtype *relative to each other* within the confines of each experiment.

* All FRAP studies listed here either used Green Fluorescent Protein (GFP) or mCherry Fluorescent Protein (ChFP). Those fluorescent proteins fused to the N-terminus of H1 are marked NTD and those fused to the C-terminus are marked CTD.

**Table 3.** Chromatin and DNA Interaction Characteristics of H1 Subtypes.

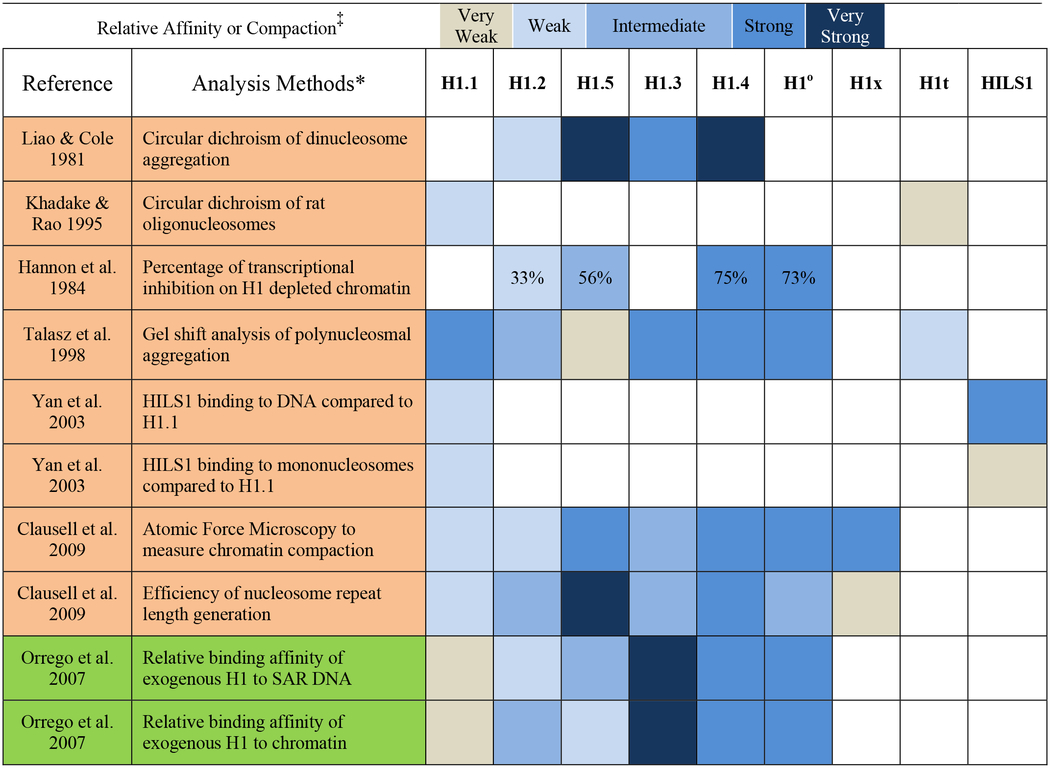

*In vitro* studies using H1 depleted chromatin are colored tan and studies using intact chromatin are colored in green.

‡ Most results are reported in relation to the other subtypes in the study. To harmonize findings from disparate reports lacking actual numeric values, I have established a color code to visualize the level of affinity or compaction described by the authors.

* No single study has evaluated all 11 subtypes, in fact there are no quantitative binding studies for H1T2 to H1-depleted oligonucleosomes or intact chromatin that this author is aware of at this time.

**Table 4.** H1 Subtype Number versus Cell Type Number.

Organism	Known Subtypes [43]	Estimated Cell Type Number
Homo sapiens	11	210 [180] 411 (including diversity of neurons) [181]
Mus musculus	11	100–150 [182]
Gallus gallus	7	100–150 [182]
Xenopus laevis	5	100–150 [182]
Nicotiana tabacum	6	> 40 [183]
Arabidopsis thaliana	3	> 40 [183]
Drosophila virilis	3	??
Drosophila melanogaster	1	??
Caenorhabditis elegans	8	??
Saccharomyces cerevisiae	1	1
Tetrahymena thermophila	1	1

## Abbreviations

bp: Base pairs
CD: Circular Dichroism
CDK: Cyclin Dependent Kinase
ChIP: Chromatin Immunoprecipitation
ChIP-seq: ChIP followed by sequencing of isolated DNA
CTD: C-terminal Domain
DDR: DNA Damage Response
ESC: Embryonic Stem Cell
FRAP: Fluorescence Recovery After Photobleaching
GD: Globular Domain
GV: Germinal Vesicle
HSP or Hsp: Heat Shock Protein
IR: Infrared Spectroscopy
KO: Knockout
NMR: Nuclear Magnetic Resonance
NRL: Nucleosome Repeat Length
NTD: N-terminal Domain
RBC: Red Blood Cells
RT-PCR: Real-Time PCR
SAR: Scaffold-Associated Region
shRNA: Short Hairpin RNA
TFE: Trifluoroethanol
